# Prenatal inflammation exacerbates hyperoxia-induced neonatal brain injury

**DOI:** 10.1186/s12974-025-03389-4

**Published:** 2025-02-28

**Authors:** Meray Serdar, Kay-Anja Walther, Markus Gallert, Karina Kempe, Stefanie Obst, Nicole Labusek, Ralf Herrmann, Josephine Herz, Ursula Felderhoff-Müser, Ivo Bendix

**Affiliations:** Department of Paediatrics I, Neonatology and Experimental perinatal Neurosciences, Centre for Translational Neuro- and Behavioural Sciences (C-TNBS), University Hospital Essen, University Duisburg-Essen, Essen, Germany

**Keywords:** White matter injury, Preterm birth, Prenatal inflammation, Postnatal hyperoxia, Oligodendrocytes, Microglia, Cytokines, Neuroinflammation

## Abstract

**Background:**

Premature born infants are at high risk to develop white matter injury (WMI). Hyperoxia and perinatal inflammation are main risk factors for preterm birth and associated brain injury. To date the majority of experimental studies have focused on isolated insults. However, clinically, WMI injury is a multifactorial disorder caused by a variety of triggers. To establish a clinically relevant rodent model of WMI, we combined prenatal inflammation with postnatal hyperoxia to investigate individual, and additive or synergistic effects on inflammatory processes, myelination and grey matter development.

**Methods:**

At embryonic day 20, pregnant Wistar rat dams received either a single intraperitoneal injection of 100 µg/ kg lipopolysaccharide (LPS) or sodium chloride. Offspring were either exposed to hyperoxia (80% O_2_) or normoxia (21% O_2_) from postnatal day 3 to 5. Animals were sacrificed immediately after hyperoxia or 6 days later, corresponding to term-equivalent age. White and grey matter development and neuroinflammatory responses were investigated at cellular and molecular levels applying immunohistochemistry, western blotting, real time PCR in brain tissues and multiplex protein expression analysis on serum samples.

**Results:**

Prenatal inflammation combined with postnatal hyperoxia resulted in reduced body weight and length in the offspring, accompanied by increased serum leptin levels at term equivalent age. The altered body parameters, like body weight, were associated with decreased brain volume, thinning of deep cortical layers and hypomyelination. As potential underlying mechanisms, we identified severe myelination deficits and an increased microglia activation associated with elevated inflammatory cytokine expression in brain tissues, while peripheral cytokine levels were reduced. Interestingly, effects on body size were mainly mediated by prenatal LPS, independent of hyperoxia, while oligodendrocyte degeneration was mainly induced by postnatal hyperoxia, independent of prenatal inflammation. However, for the majority of pathological changes, including brain size, myelination deficits, microglia activation and inflammatory cytokine expression, additive or synergistic effects were detected.

**Conclusion:**

Prenatal inflammation combined with postnatal hyperoxia results in aggravated myelination deficits and inflammatory responses compared to single insults, making it an ideal model to improve our understanding of the complex pathophysiology underlying WMI and to evaluate urgently needed therapies.

**Supplementary Information:**

The online version contains supplementary material available at 10.1186/s12974-025-03389-4.

## Background

Representing 10% of newborns in Western countries, preterm born infants are the largest paediatric patient cohort [1, 2]. In particular, extremely preterm born infants (< 28 weeks of gestation) are at high risk of neurodevelopmental impairment, caused by encephalopathy of prematurity (EoP), which is associated with long-lasting disabilities such as motor-cognitive deficits and neuropsychiatric problems [3–6]. A major hallmark of EoP is diffuse white matter injury (WMI) associated with altered grey matter development [5, 7]. The pathophysiological mechanisms underlying WMI include degeneration and an impaired differentiation of immature oligodendrocytes, accompanied by pronounced neuroinflammatory responses, such as microglia activation [8–11]. Clinical and experimental studies have identified several noxious stimuli that contribute to WMI, including growth restriction, pain, stress and most importantly, perinatal inflammation and disturbances in oxygen supply, i.e. hypoxia or hyperoxia [12–15]. This has led to the “multiple hit hypothesis”, assuming that prenatal factors sensitize the developing brain, rendering it more vulnerable to injury from secondary insults in the early postnatal period [16–18]. To date, there is no effective therapy for the treatment of WMI for prematurely born infants. Furthermore, the majority of experimental studies have focused on either single insults [6, 19–21] or a combination of postnatal insults [13]. Therefore, investigating the combined effects of prenatal and postnatal insults in clinically relevant animal models are of high importance to better understand the complex pathophysiology of WMI and to evaluate targeted treatment strategies.

Several animal models have been developed to mimic WMI such as those induced by postnatal inflammation [20, 22], hypoxia [23, 24], and hyperoxia [21, 25–28]. In our previous work, we have shown that postnatal inflammation, induced by Lipopolysaccharide (LPS) injection combined with postnatal hyperoxia results in subacute hypomyelination deficits comparable to single insults [13]. However, the target mechanisms differed (oligodendrocyte cell death vs. differentiation) [13]. Furthermore, the inflammatory insult was set at postnatal age, whereas the development of WMI in preterm infants is often related to prenatal infection/inflammation [12, 19]. The time point of LPS application appears to be particularly important in sensitizing the brain to a second insult. As such, postnatal LPS application showed a neuroprotective effect on induced injury by secondary hypoxia [29], while LPS application during pregnancy aggravated WMI, demonstrated by increased hypomyelination and altered microglia morphology [30–32]. In addition to oxygen deprivation, hyperoxia is a frequent clinical condition, not only due to physiological differences between in utero and postnatal oxygen tension but also due to the additional required oxygen supply in a significant proportion of preterm infants [33]. Systematic comparisons between single and combined insults have rarely been assessed in previous work, hampering conclusions about potential combined effects.

In this study, we combined two clinically relevant factors contributing to WMI: maternal inflammation induced by LPS application in rat dams at late gestational age (E20) corresponding to the last trimester in humans [34, 35]. Offspring were exposed to hyperoxia (80% O_2_) from postnatal day 3 (P3) to P5, aligning with the brain development stage of a preterm infant at 26–28 weeks of gestation [34, 36]. Outcomes were assessed at term-equivalent age of P11 equivalent to 40 weeks of gestation in humans [35, 36]. We hypothesized that prenatal inflammation exacerbates hyperoxia-induced WMI and associated neuroinflammation.

## Material and methods

### Animal experiments, group allocation and tissue collection

**Fig. 1:**
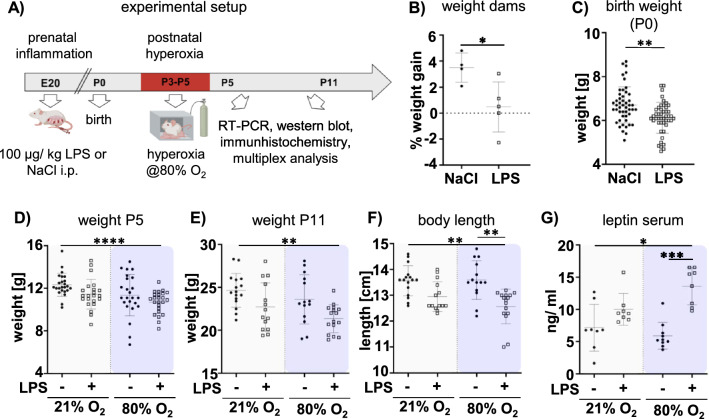
Prenatal inflammation combined with postnatal hyperoxia leads to growth restriction and disturbed metabolism. **A** Experimental setup (created wit BioRender): Dams received a single intraperitoneal (i.p.) injection of 100 µg/ kg LPS or NaCl at embryonic day 20 (E20). Newborn pups were exposed to normoxia (21% O_2_) or hyperoxia (80% O_2_) for 48 h from postnatal day P3 to P5. Analyses were performed either immediately after hyperoxia at P5 or at term-equivalent age at P11. **B** Alterations in dam weight was recorded within 24 h after LPS application (n = 4–5 dams/ group). **C**–**E**) The offspring’s body weight was recorded daily and is shown here for relevant ages P0, P5 and P11. **F** At P11 the body length (head to tail) was measured. **G** Leptin protein levels were analysed in serum samples by multiplex protein expression analyses. n = 8–10 rats/group at P5, n = 14–16 rats/group at P11. *p < 0.05, **p < 0.01, ***p < 0.001

Experiments were performed in accordance with the Animal Research Reporting of In Vivo Experiments (ARRIVE) guidelines with governmental approval by the State Agency for Nature, Environment and Consumer Protection North Rhine-Westphalia, Germany. Wistar rats were bred in house and kept under a 12 h light/ dark cycle with food and water ad libitum. To mimic a clinically relevant condition of WMI, we designed an experimental setup combining prenatal inflammation and postnatal hyperoxia (Fig. 1A). Time-pregnant Wistar rats received a single intraperitoneal (i.p.) injection of 100 µg/ kg LPS (E.coli O55:B5, LL-423, Biotrend, Germany), dissolved in 0.9% sodium chloride (NaCl) at 50°C for 10 min, aliquoted and stored at −20°C, or 0.9% NaCl (vehicle) at embryonic day 20 (E20). Previous studies showed that maternal inflammation induced by LPS injection at late gestational age leads to alterations in fetal cytokine production, increased leukocyte recruitment to fetal membranes and more organ infiltration in the fetus, supporting a heightened inflammatory state in the fetus [37]. Furthermore, increased levels of pro-inflammatory cytokines were detected particularly in the brain, which persist until birth [38]. Therefore, we refer to the term prenatal inflammation in the present experimental setting. Dams were weighed daily up to two days after birth. Pups body weight was recorded every day until sacrifice at postnatal day 5 (P5) and postnatal day 11 (P11), respectively. According to our previous work [25], P3 pups were exposed to hyperoxia (80% O_2_) for 48 h in an oxygen chamber (OxyCycler, BioSperix, Lacona, NY, USA). Appropriate controls were kept under normoxic conditions (21% O_2_). In this experimental setting we did not observe mortality due to prenatal inflammation and/ or postnatal hyperoxia in pups and rat dams, respectively. In total 94 pups derived from nine litters were enrolled in this study. Sixty pups (28 female, 32 male) were used for analyses of subacute white matter injury at P11. Another set of 34 animals (14 female, 20 male) was analysed at P5. All pups were randomly assigned to the following experimental groups: NaCl + 21% O_2,_ LPS + 21% O_2,_ NaCl + 80% O_2,_ LPS + 80% O_2_.

At the corresponding analyses time points (P5 or P11) pups were deeply anesthetised with chloralhydrate; body length was determined followed by transcardial perfusion with ice-cold PBS. Brains without cerebellum were divided into hemispheres. The right hemisphere was used for histological and immunohistochemistry analyses by immersion fixation in 4% paraformaldehyde (PFA) overnight and paraffin-tissue embedding. For real time PCR and western blot analysis the left hemisphere was snap frozen in liquid nitrogen and stored at −80°C until further processing.

### Histology and immunohistochemistry analyses

Analyses were performed on 7 µm paraffin tissue sections taken from hippocampal (− 3.72 ± 0.7 mm) and striatal (− 0.6 ± 0.3 mm) tissue levels. Hemisphere volumes was determined in cresyl-violet stained tissue sections taken from bregma level − 4.1 ± 0.2 to − 0.5 ± 0.2 mm at a distance of 140 µm. Volume was calculated by summing up part volumes between tissue sections, which were calculated by the following formula: section area*distance to the next section [39].

For immunohistochemistry analyses, tissue sections were stained according to previous studies [13, 21]. Myelination and oligodendrocyte differentiation capacity were analysed by staining of adenomatous polyposis coli, clone CC1 positive (referred as CC1, mature oligodendrocytes; P5 and P11) and myelin basic protein (MBP; P11) combined with oligodendrocyte transcription factor 2 (Olig2). Oligodendrocyte proliferation was evaluated by co-staining of Ki67/ Olig2 and cellular degeneration was determined by staining of DNA fragmentation using terminal transferase dUTP nick end labeling (Tunel) according to the manufactures’ protocol (In situ Cell Death Detection Kit, Roche, Switzerland) in combination with Olig2. Microglia activation was assessed in co-staining of ionized calcium-binding adaptor protein 1 (Iba1) and macrosialin (CD68) [40]. Cortical thickness in the deep cortical layers was analysed in tissue sections stained for the transcription factor T-box brain 1 (TBR1). Briefly, sections were deparaffinised and rehydrated followed by antigen-retrieval in pre-heated 10 mM sodium-citrate-buffer (pH 6.0) for 30 min. After blocking with 1% bovine serum albumin, 0.3% cold-fish-skin-gelatine in 1% Tween-20 in phosphate-buffered saline (PBS-T), slides were incubated with the primary antibody over night at 4°C. The following primary antibodies were used: rabbit anti-Olig2 (1:100, Millipore, Germany), mouse anti-CC1 (1:100, Calbiochem, Germany), rat anti-MBP (1:100, abcam, Great Britain), rabbit anti-TBR 1 (1:100, abcam), rabbit anti-Iba (1:1000, WAKO, USA), mouse anti-CD68 (1:100, Bio Rad, USA) and mouse anti-Ki67 (1:100, abcam). Antibody binding was visualised by incubation with appropriate Alexa Fluor 488, Alexa Fluor 555 or Alexa Fluor 647 conjugated secondary antibodies (anti-rat/mouse or rabbit, all 1:500 Thermo Fisher, Germany) for 2 h at room temperature. Nuclei were counterstained with 4′, 6-Diamidin-2-phenylindol (DAPI, 100 ng/ml; Molecular Probes, USA).

Sections were assessed by confocal imaging (A1plus, Eclipse Ti, with NIS-Elements AR software, Nikon, Germany, four laser lines (laser diode 405 nm, Ar laser 514 nm, G-HeNe laser 543 nm, Rn laser 639 nm). Confocal z-stack (7.5 µm thickness, z-plane distance 1.5 µm) large-scale images of the entire hemispheres were acquired with the 10 × objective. From each animal two slides were assessed, and data were analysed by investigators blinded to treatment. Nine regions of interests (ROI, each 302.5 µm^2^; three ROIs white matter, three ROIs cortex and three ROIs thalamus, Suppl. Fig. S1) were analysed. For structural analysis of MBP fibres, three ROIs of the white matter (Suppl. Fig. S1, yellow boxes) were acquired with the 20 × objective. All images were converted into maximal intensity projections. Unbiased software-based object counting was used to assess the number TBR1^+^cells. The number of CC1^+^, KI67^+^ and Tunel^+^ was determined manually and related to the ROI area. The percentage of Iba1^+^ and MBP^+^ area normalized to ROI areas (Iba-1) or hemisphere areas (MBP) was quantified as measures of microglia cell density and the degree of myelination, respectively. The percentage of CD68^+^ area normalized to the Iba1^+^ area was investigated as a measure of activated microglia. Morphological analysis of Iba1^+^ microglia was performed semi-automatically with NIS elements software. As a measure of morphological changes elongation was quantified, i.e. reduced values reflect a more amoeboid morphology and increased values can be interpreted as longer processes, i.e. more ramification/ decreased activation [41, 42]. For structural analysis of myelin fibres, 3D images of MBP stained tissue sections were converted to TIFF-images followed by analysis with the adapted DiameterJ plugin of ImageJ (National Institutes of Health, Java 1.4.0) [43]. Thickness of the deep cortical layer, stained by TBR1, was demarcated manually and related to the section area of the whole hemisphere.

### Protein and RNA isolation

Protein and RNA were isolated from left hemispheres with the TRIzol procedure (Thermo Fisher, Germany) using the QIAzol reagent (Qiagen, Germany). Briefly, tissue samples were homogenized in QIAzol Reagent followed by the addition of chloroform to separate the homogenate into different phases. The clear upper aqueous phase was used for RNA precipitation. The precipitated RNA was washed with ethanol, subsequently air-dried, and resuspended in RNAse-free water. RNA yield was determined with the NanoDrop Spectrophotometer (Peqlab, Germany). Ethanol was added to the phenol–chloroform phase and centrifuged, the supernatant was used for protein precipitation by adding isopropanol. Precipitated protein samples were washed three times with guanidine hydrochloride, followed by an ethanol washing step and air-drying of the pellet. Pellets were dissolved in 1% sodium dodecyl sulfate (SDS) for 2 days at 40°C. Protein as well as RNA were stored at − 80°C until further use.

### Western blot

After quantification of the protein concentration using the Pierce BCA assay (Thermo Scientific, USA) 20 µg protein per lane were separated on 12.5% SDS polyacrylamide gels including 2,2,2,-trichlorethanol for visualization of total protein abundance [44]. Separated proteins were transferred to nitrocellulose membranes (0.2 µm, Amersham) at 4°C overnight and equal loading and transfer of proteins was further confirmed by membrane staining with Ponceau S solution (Sigma Aldrich). Nonspecific binding was blocked by incubation in 5% non-fat dry milk powder (Cell Signaling) and 0.1% Tween-20 in tris-buffered saline (TBS) followed by incubation with the primary antibodies Myelin associated glycoprotein (MAG; 1:1000, abcam) and 2′, 3′-Cyclic-nucleotide-3′-phosphodiesterase (CNPase; 1:2000, Millipore) overnight at 4°C. Membranes were incubated with appropriate peroxidase-conjugated secondary antibodies (all 1:5000, Dako) in blocking solution at room temperature for 1 h followed by chemiluminescent detection with the enhanced chemiluminescence prime western blotting detection reagent (Amersham, GE Healthcare Life Science, USA). The total amount of transferred proteins and specific antibody-labelled proteins were visualised and analyzed with the ChemiDocXRS + imaging system and ImageLab software (Bio-Rad). The target protein expression was normalised to whole protein content and the control group NaCl + 21% O_2_.

### mRNA expression analysis

First strand complementary DNA was synthesized using 1 μg of total RNA and TaqMan reverse transcription reagents (Applied Biosystems/Thermo Fisher Scientific). PCR amplification was performed in 96 well optical reaction plates for 40 cycles with each cycle at 94°C for 15 s and 60°C for 1 min using the StepOnePlus Real Time PCR system (Applied Biosystems/Thermo Fisher Scientific). PCR products were quantified by the use of fluorogenic reporter oligonucleotide probes (Suppl. Table 1). Analysis was performed in duplicates and target gene expression was quantified according to the 2^−ΔΔCT^ method [45] with animals of the NaCl + 21% O_2_ group serving as controls.

### Multiplex-analysis of serum samples

Blood was collected from the right atrium prior to perfusion, collected and centrifuged at 3500 × *g* for 7 min at 4°C. The supernatant was frozen at − 80°C until further analysis. Serum samples were analyzed for the abundance of TNFα, IL 6, IL 10, IL 4, and Leptin performing an MSD multiplex screening assay (Meso Scale Discovery, USA). Sampling and assay conduction were exactly performed as described in the manufacturers’ manual. Briefly, MSD Multiplex Assay allows the simultaneous detection of multiple analytes in a single sample using electrochemiluminescent (ECL) detection. Capture antibodies, specific to each analyte were coated on a 96-well-plate. Binding of target molecules within the sample to capture antibodies was visualised by incubation with ECL-labeled secondary antibodies. In the present study, 25 µl of serum was required to achieve signals above the detection limit. ECL-signals were analysed with the MESO QuickPlex SO 120MM system using the Methodical Mind software (Meso Scale Discovery, USA).

### Statistical analysis

Results are expressed as scatter plots with individual data points, including median values, the 25th and the 75th percentiles. Statistical analyses were performed using the GraphPad Prism 9.0 software package (GraphPad Software). Two-way ANOVA was applied to determine main and interaction effects between treatment groups (Suppl. Table S1). For intergroup comparisons, data were tested for Gaussian distribution and analysed by one-way ANOVA followed by Bonferroni's multiple comparison test for parametric data or Kruskal–Wallis test with Dunn's multiple comparison test for non-parametric data. We defined synergistic effects between treatment groups when single insults did not show differences compared to controls, but the combination of insults resulted in significant group differences [46]. This was also confirmed by significant interaction effects in two-way-ANOVA analyses (Suppl. Table S2–4). Additive effects were defined when the combination resulted in larger group differences compared to effects by single insults, which should, however, not be larger than the sum of single effects. Principal component analysis (PCA) was performed using R packages FactoMineR [47]. Depending on data distribution, correlation analyses were either performed with Pearson tests (parametric) or Spearman tests (non-parametric).

## Results

### Prenatal inflammation combined with postnatal hyperoxia decreases body growth

Severe prenatal inflammation, linked to preterm birth and growth restriction [48–51], combined with oxygen excess, remain the most important risk factors for WMI [33, 52, 53]. To model this, we combined prenatal inflammation induced by i.p. injection of LPS with postnatal hyperoxia between P3 and P5 (Fig. 1A). While litter size was not affected (NaCl: 10.8 ± 1.5; LPS: 11 ± 1.9), prenatal LPS exposure induced a significant reduction in the weight gain of dams 24 h after injection (NaCl: 3.5 ± 1.1%; LPS: 0.48 ± 1.92%, Fig. 1B). Interestingly, prenatal LPS exposure also led to growth restriction in the offspring revealed by a significantly lower body weight of pups at P0 (NaCl: 6.6 ± 0.98 g; LPS: 6.1 ± 0.69 g, Fig. 1C). Reduced body weights in LPS offspring persisted until term equivalent age, i.e. P11 (Fig. 1C–E). Even though the offspring’s weight was also slightly reduced by postnatal hyperoxia, the most pronounced effects were detected in pups from LPS-treated dams and additional hyperoxia (Fig. 1C–E). Reduced body weights in offspring of LPS-treated dams were accompanied by decreased body length, which was slightly reduced by postnatal hyperoxia (Fig. 1F). Nevertheless, the main factor contributing to reduced growth was prenatal LPS exposure (F = 11.78, p = 0.0011 Suppl. Table S2). Growth restriction, induced by prenatal factors is often associated with metabolic disturbances, including alterations in leptin signalling [54]. Therefore, we quantified the amount of circulating leptin levels, which in addition to its endocrine function in metabolism is also required for normal brain development by regulating neurogenesis and circuit formation [55]. Interestingly, while single postnatal hyperoxia had limited effects on leptin levels in the offspring, prenatal LPS increased serum leptin levels, which were further elevated in the combined setting (Fig. 1G), indicating a synergistic effect, as revealed by a significant interaction between both single insults (F = 6.383, p = 0.0172, Suppl. Table S2).

### Double hit of prenatal inflammation and postnatal hyperoxia results in reduced brain volume and decreased deep cortical layer thickness

**Fig. 2:**
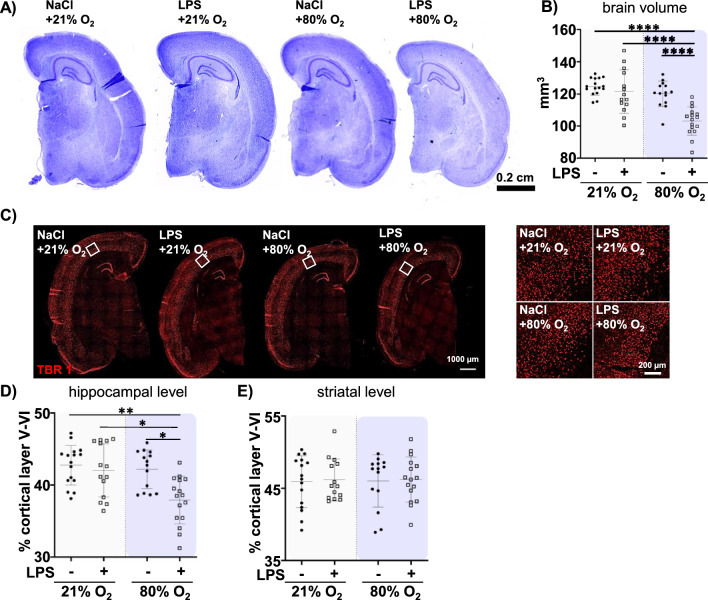
The combination of prenatal inflammation and postnatal hyperoxia leads to a reduced brain volume and deep cortical layer thinning at P11. After prenatal inflammation at E20 pups were exposed to postnatal normoxia (21% O_2_) or hyperoxia (80% O_2_) from P3 to P5 and analysed at P11. **A**, **B** Hemisphere volume were determined on cresyl-violet stained sections taken from bregma level − 4.1 ± 0.2 to − 0.5 ± 0.2 mm with a distance of 140 µm between sections. **C** Cortical thickness of layer V and VI was analysed in TBR1 stained tissue sections. White boxes illustrate the deep cortical layer V–VI depicted on the right side. **D**, **E** The ratio of the TBR1 positive deep cortical layer related to the whole hemisphere area was quantified at the hippocampal (3.72 ± 0.7 mm, **D**) and striatal (− 0.6 ± 0.3 mm, **E**) level. n = 14–16 rats/ group. *p < 0.05, ***p < 0.001, ****p < 0.0001

To assess whether reduced body weight and length were associated with differences in brain growth, the hemisphere volume was quantified in cresyl-violet stained tissue sections at P11 (Fig. 2A, B). While only slight reductions were observed with single insults, we detected a remarkable decrease in brain volume in the combined insult group (Fig. 2B), suggesting synergism between single insults as demonstrated by a significant interaction (F = 8.370, p = 0.0054, Suppl. Table S2). A reduction in brain volume has recently been linked to decreased cortical thickness in extremely preterm born infants [56]. Therefore, we quantified the thickness of the deep cortical layers V-VI by immunohistochemistry for TBR1 (Fig. 2C). While only slight effects were detected in single treatment groups, the combination of prenatal inflammation and hyperoxia caused a significant reduction in the proportion of the TBR1 positive layer at the hippocampal level indicating synergy supported by a significant interaction between both single insults (F = 4.843, p = 0.0319, Suppl. Table S2 Fig. 2D). No differences were observed at the striatal level (Fig. 2E). Furthermore, neither the cellular density of TBR1 positive neurons in the total TBR1^+^ cortical layer (Suppl. Figure 2A) nor the global expression of the post-mitotic marker NeuN in total hemisphere protein lysates (Suppl. Figure 2B) were modulated, suggesting a selective atrophy of the cortical layers V-VI, near the white matter.

### Prenatal inflammation and postnatal hyperoxia differently affect oligodendrocyte survival and maturation

**Fig. 3:**
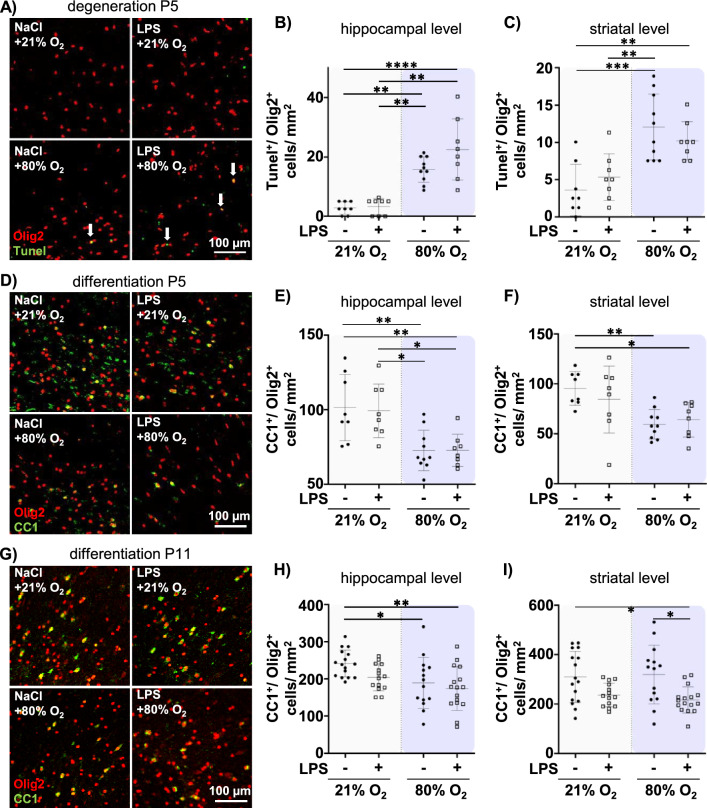
Prenatal inflammation and postnatal hyperoxia have a different impact on the number of degenerating and mature oligodendrocytes in a time point- and region-specific manner. Pups from dams with prenatal inflammation and additionally exposed to postnatal normoxia (21% O_2_) or hyperoxia (80% O_2_) at P3 for 48 h were analysed immediately after hyperoxia at P5 or at term-equivalent age at P11. **A** Oligodendrocyte degeneration was assessed in Olig2 (red)/ Tunel (green) co-staining at P5. Arrows indicate Olig2/ Tunel co-localizations (yellow). **B**, **C** The number of double positive cells was analysed in the white matter at the hippocampal (3.72 ± 0.7 mm, **B**) and striatal (− 0.6 ± 0.3 mm, **C**) level. **D**–**I** The number of mature oligodendrocytes was analysed in tissue sections stained for Olig2 (red) and CC1 (green). Olig2/ CC1 double positive cells were quantified at P5 (**E**, **F**) and P11 (**H**, **I**). Example images in **A**, **D** and **G** are derived from the cingulum of the white matter region (Suppl. Figure 1). n = 8–10 rats/group at P5, n = 14–16 rats/group at P11. *p < 0.05, **p < 0.01, ***p < 0.001

Proper neurodevelopment depends on the support of oligodendrocytes. However, in the premature brain, the majority of oligodendrocytes is still immature, making them particularly vulnerable to pre- and postnatal insults such as inflammation and hyperoxia [8, 10, 13, 21, 22]. The most prominent hallmarks of WMI are degeneration and impaired differentiation of immature oligodendrocytes, resulting in disturbed myelination, a major determinant of long-term neurodevelopmental deficits [21, 27, 57]. To assess a potential relation between disturbed cortical neuronal development (Fig. 2C–E) and oligodendrocyte responses, we focused our analyses on white matter regions in close proximity to the degenerated cortical layer V-VI (Suppl. Figure 1A, B). Quantification of Tunel^+^/Olig2^+^ and mature CC1^+^ cells revealed a marked increase in oligodendrocyte degeneration and a reduction in mature oligodendrocytes immediately after hyperoxia at P5, which was, however, independent of prenatal LPS exposure (Fig. 3, main effect hyperoxia, p < 0.001, 3B) F = 65.53; 3C) F = 30.11; 3E) F = 23.38; 3F) F = 12.51, Suppl. Table S2). Similar regulations were observed in other brain regions, i.e. cortex, thalamus and striatum (Suppl. Figure 3A–D). To determine whether the reduction in mature CC1^+^ cells was not only due to cell degeneration but also to decreased generation of new oligodendrocytes, we quantified oligodendrocyte proliferation (Suppl. Figure 4 A). Except of slight reductions by postnatal hyperoxia in the thalamus and striatum, no differences were detected in other brain regions (Suppl. Figure 4 B, C) excluding compensatory effects by oligodendrocyte proliferation at P5.

To assess whether the acute loss of mature CC1^+^ oligodendrocytes at P5 translates into the subacute phase at term equivalent age, we further quantified CC1^+^/Olig2^+^ cells at P11 (Fig. 3G–I). Between P5 and P11 regulation of CC1^+^ cell numbers changed depending on the insult. As such, single postnatal hyperoxia was still associated with reduced numbers in the white matter at the hippocampal level, again independent of prenatal LPS exposure (Fig. 3G, H). However, prenatal LPS exposure had a stronger effect at P11 compared to P5, especially in the white matter at the striatal level, which was not further modulated by postnatal hyperoxia (Fig. 3F, I). While we detected a similar impact of prenatal LPS in the cortex, single hyperoxia led to significant reductions of CC1^+^ oligodendrocytes in the thalamus and striatum (Suppl. Figure 5 A, B). Taken together, these data indicate that postnatal hyperoxia leads to acute oligodendrocyte degeneration associated with reduced numbers of mature oligodendrocytes, whereas prenatal LPS is associated with a secondary reduction in mature oligodendrocytes at P11 indicating impaired oligodendrocyte maturation. Therefore, both, oligodendrocyte degeneration and maturation deficits may lead to disturbed myelination.

### Prenatal inflammation exacerbates myelination deficits caused by postnatal hyperoxia

**Fig. 4:**
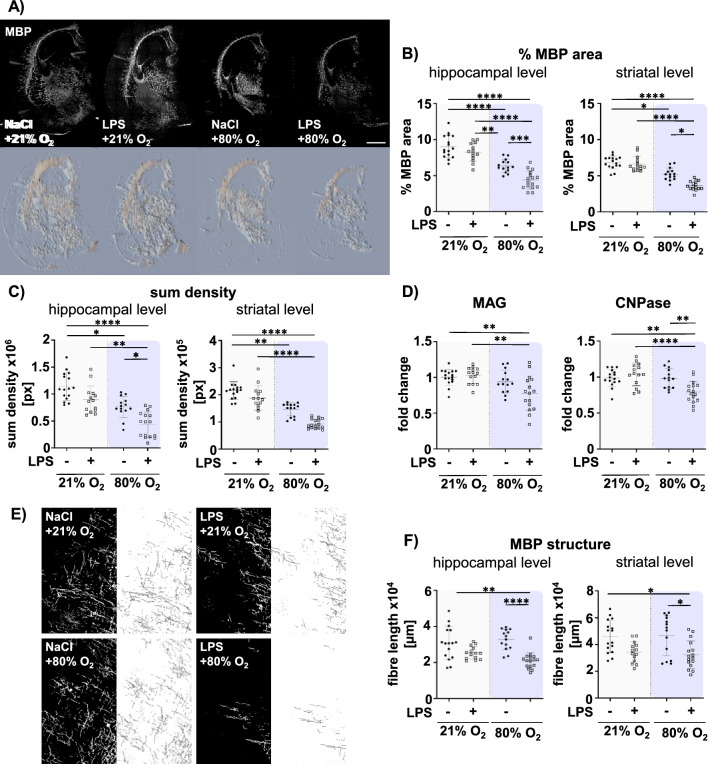
The double-hit of prenatal inflammation and postnatal hyperoxia induces pronounced myelination deficits associated with structural changes. Myelination was analysed at term equivalent age P11, i.e. 6 days after postnatal normoxia (21% O_2_) or hyperoxia (80% O_2_) via immunohistochemistry and real time PCR. **A** Representative images of MBP (myelin basic protein) staining (upper: immunohistochemistry MBP-staining; lower: sum density MBP-staining). **B** The MBP positive area related to the whole hemisphere area was analysed at the hippocampal (3.72 ± 0.7 mm) and the striatal level (− 0.6 ± 0.3 mm). **C** The same images were used to analyse the sum pixel density of MBP staining normalized to the hemisphere. **D** mRNA expression of MAG and CNPase from whole hemisphere was quantified by RT-PCR. **E** Representative images in are derived from the deep cortical white matter and the appropriate skeletonized pictures. **F** Structural formation of MBP fibre was evaluated using by the adapted DiameterJ plug Image J enabling skeletonization of images from MBP staining (**E**). The fibre length was quantified in 3 ROIs of the white matter (Suppl. Figure 1). n = 14–16 rats/ group. Scale bar (white) 1000 µm and scale bar (black) 100 µm. *p < 0.05, **p < 0.01, ****p < 0.0001

Mature oligodendrocytes are the most important cell population for developmental myelination processes in the postnatal period [58]. To determine whether the detrimental effects on mature oligodendrocytes were also associated with myelination deficits, we assessed the expression levels of key components of the myelin sheath (i.e. MBP, MAG and CNPase) and evaluated the structural integrity of myelin fibres. Confirming previous work [21, 25, 27, 59], postnatal hyperoxia resulted in a marked hypomyelination revealed by a reduction of MBP positive area (Fig. 4A, B) and sum pixel density (Fig. 4A, C). Furthermore, prenatal inflammation also induced a reduction in myelination, although the effects were smaller compared to postnatal hyperoxia (Fig. 4A–C). Importantly, the combination of both showed a stronger decrease in MBP expression compared to single insults, suggesting additive effects (Fig. 4A–C). In addition to MBP, MAG and CNPase are central components of the myelin sheath [60]. We determined a similar reduction of MAG and CNPase expression in the double hit compared to controls and single hits (Fig. 4D, Suppl Fig. 5 D). Notably, compared to the regulation of MBP expression, which revealed a reduction by both single insults, MAG and CNPase RNA expression was not strongly altered by either single hit (Fig. 4D). However, both insults act synergistically, demonstrated by a pronounced reduction of MAG and CNPase expression in the combined setting (Fig. 4D).

MBP and MAG are essential for compact myelin development and wrapping of axons supporting their integrity and thus proper neurodevelopment [61, 62]. Therefore, we also quantified the structural organisation of myelin fibres, i.e. fibre length and intersections in high resolution images of MBP stained tissue sections (Fig. 4E–F. Consistent with the reduction in MBP expression induced by prenatal LPS combined with postnatal hyperoxia exposure (Fig. 4A-C), fibre length and the number of intersections were significantly decreased at both anatomical brain levels (Fig. 4F, Suppl. Fig. S5C). These morphological changes were, however, mainly driven by prenatal LPS exposure (main effect fibre length, hippocampal level F = 29.41, p < 0.0001; striatal level F = 18.74, p < 0.0001, Suppl. Table S3), while single postnatal hyperoxia had no effect on these parameters (Fig. 4F, Suppl. Figure 5C).

### The double hit of prenatal LPS plus postnatal hyperoxia induces microglial activation associated with increased inflammatory cytokine expression

**Fig. 5:**
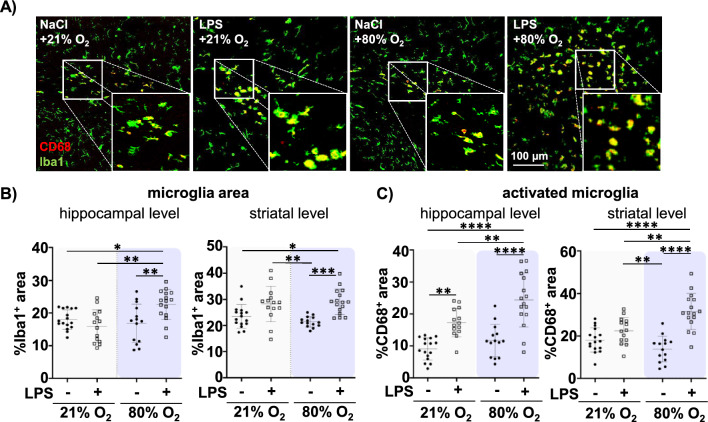
Prenatal inflammation combined with postnatal hyperoxia lead to microglia activation at P11. Prenatal inflammation was induced at E20 followed by postnatal normoxia (21% O_2_) or hyperoxia (80% O_2_) at P3 for 48 h and brains were investigated at P11. **A** Microglia activation was analysed by CD68 (red) / Iba1 (green) co-staining in three regions of white matter (Suppl. Figure 6C); representative images are derived from cingulum White boxes reveal high magnification images of the staining to show morphological changes. **B** As a measure of microglia density the percentage of Iba1 positive area was quantified in the white matter of the hippocampal (3.72 ± 0.7 mm, left) and striatal level (−0.6 ± 0.3 mm, right). **C** Activation of microglia was assessed by quantification of the percentage of CD68 positive area from the total Iba1 positive area in hippocampal (left) and striatal level (right). n = 14–16 rats/ group, *p < 0.05, **p < 0.01, ***p < 0.001, ****p < 0.0001

Developmental WMI-associated myelination deficits have been largely attributed to neuroinflammatory responses in which microglia play a critical role [63, 64]. Therefore, we quantified the expression of the microglia-specific marker Iba1 and analysed the proportion of the CD68 positive area of the total Iba1 positive area at P5 (Suppl. Figure 6) and P11 (Fig. 5) in the white matter (Suppl. Figure 6 C). While we found no significant group differences at P5 (Suppl. Fig. S6 A, B), single prenatal LPS induced a slight increase of Iba1 immunoreactivity at the striatal level at P11 (Fig. 5B, C), which was not further modulated by postnatal hyperoxia (Fig. 5B, C). However, at the hippocampal level we determined synergistic effects of both single insults, as neither single insult led to changes in the Iba1 positive area, whereas a significant upregulation was observed in the combined setting of prenatal inflammation and postnatal hyperoxia (i.e. significant interaction: F = 10.46, p = 0.0020 (Suppl. Table S3, Fig. 5B). Similar combined effects were detected for microglia activation assessed by quantification of CD68 immunoreactivity on Iba1^+^ cells, described to be upregulated upon microglia activation [65] (Fig. 5C). While prenatal LPS exposure enhanced the proportion of activated CD68^+^ microglia, additional postnatal hyperoxia led to a much stronger response, significantly different from single prenatal LPS and single hyperoxia treatment, both at the hippocampal and striatal level (Fig. 5A–C). A synergistic effect was particularly evident at the striatal level, revealed by a significant interaction between both single insults (F = 14.47, p = 0.0004, Suppl. Table S3). Even though CD68 indicates microglial activation [66], it does not provide information about morphological changes, which are an additional major hallmark of microglia activation [65]. Morphological changes were analysed by automated unbiased software-based quantification of cellular elongation, revealing that the combined setting of prenatal LPS and postnatal hyperoxia exposure led to the most pronounced reduction in elongation, albeit the main driver in this analysis parameter seems to be LPS (main LPS effects: hippocampal level F = 23.54, p < 0.0001; striatal level F = 29.74, p < 0.0001).

**Fig. 6:**
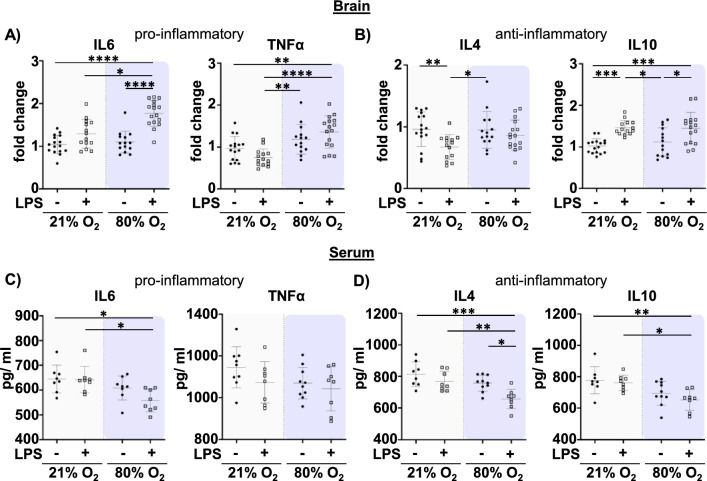
The double hit of prenatal inflammation plus postnatal hyperoxia leads to pronounced changes in inflammatory cytokine levels in the brain and the circulation at P11. After LPS application at E20 pups underwent normoxia (21% O_2_) or hyperoxia (80% O_2_) at P3 for 48 h. Analysis was performed at term equivalent age P11. **A**, **B** mRNA expression of pro- (IL6, TNFα) and anti-inflammatory (IL4, IL10) cytokines was analysed in brain tissue lysates. **C**, **D** Serum samples were analysed by multiplex protein expression analysis. n = 14–16 rats/ group for **A** and **B**, n = 8–10 rats/ group for **C** and **D**. *p < 0.05, **p < 0.01, ***p < 0.001, ****p < 0.0001

To determine whether increased microglia activation was also associated with alterations of inflammatory cytokine expression, we performed real time PCR in brain tissue lysates and multiplex analysis in serum samples for typical pro- and anti-inflammatory cytokines at P5 and P11. While, no group differences were observed at P5 (Suppl. Figure 7A–D), we detected a prominent upregulation of the pro-inflammatory cytokines IL6 and TNFα by prenatal inflammation and additional postnatal hyperoxia in brain tissue lysates (Fig. 6A). Similar to microglia activation, both single insults seem to act synergistically in the case of IL-6 expression, i.e. slightly increased expression levels induced by prenatal inflammation were strongly enhanced by postnatal hyperoxia, which by itself did not change IL6 expression (significant interaction: F = 8.410, p = 0.0053, Suppl. Table S3, Fig. 6A). Except of a slight decline by prenatal LPS exposure, no further group differences were observed for IL4 (Fig. 6B). However, we observed an upregulation for the anti-inflammatory cytokine IL10 in pups of prenatal inflammation, which was independent of postnatal hyperoxia (main effect LPS: F = 27.41, p < 0.0001, Suppl. Table S3, Fig. 6B). To check whether changes in the CNS were associated with different peripheral immune responses, we analysed serum samples. Here, we observed the opposite regulation, as shown by a reduction of pro- and anti-inflammatory cytokines in the combined setting, which were mainly driven by postnatal hyperoxia (main effect hyperoxia: IL6 F = 11.24, p = 0.0023; TNFα F = 4.199, p = 0.0493; IL4 F = 14.70, p = 0.0006; IL10 F = 15.05, p = 0.0005, Suppl. Table S3, Fig. 6C, D).

### Combined hits of prenatal inflammation and postnatal hyperoxia induce a unique EoP phenotype, clearly distinguished from single insults

**Fig. 7:**
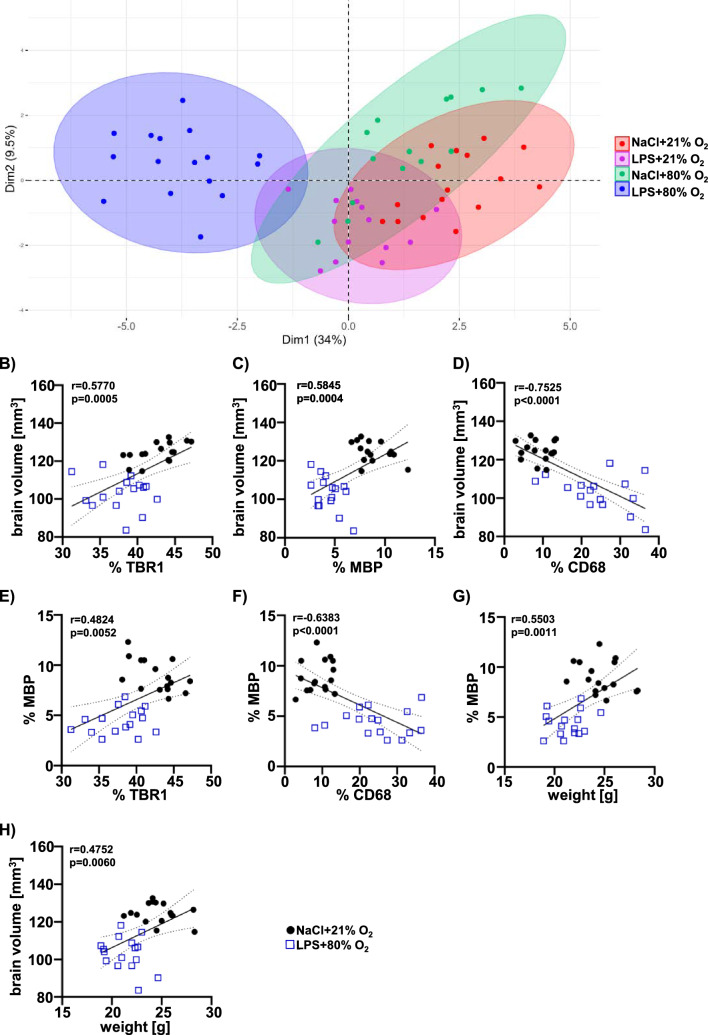
Pups exposed to prenatal inflammation plus postnatal hyperoxia clearly separate from single insults and controls. To visualise overlapping and combinational effects of the investigated noxious insults postnatal hyperoxia (80% O_2_) and prenatal LPS injection, a principal component analysis (PCA) was performed based on 21 parameters (sex, weight P5, weight P11, body length, brain volume, MAG protein level, MAG gene level, IL6, TNFα, IL10, IL4; hippocampal and striatal level: % MBP, sum density MBP, % CD68, % Iba1, fibre length, intersection, % TBR1; serum: leptin, IL6, TNFα, IL10, and IL4). n = 14–16 rats/ group. **B**-**H**) Based on body weight and histological data (hippocampal level) at term-equivalent age (P11), correlation analyses were performed for key features of WMI injury, i.e. body weight, brain volume, cortical thickness (%TBR1), myelination (%MBP) and microglial activation (%CD68). Correlation coefficients (r) and p-values are indicated in the graphs. n = 16 rats/ group

Despite individual treatment effects, observed for certain pathophysiological hallmarks (e.g. brain volume, hypomyelination and microglia activation), principal component analysis including all analysed parameters of the present study demonstrated a clear separation of the combined treatment group from controls, while single insults of prenatal inflammation and postnatal hyperoxia were less separated from controls (Fig. 7). This is supported by correlation analyses for immunohistochemistry data at the hippocampal level, revealing significant positive correlations between brain volume and cortical thickness (Fig. 7B), brain volume and myelination (Fig. 7C), myelination and cortical thickness (Fig. 7E), myelination and body weight (Fig. 7G); and between brain volume and body weight (Fig. 7H). The relation between microglia activation and brain volume as well as myelination was shown by significant negative correlations (Fig. 7D,F). Similar results were obtained for correlation analyses at the striatal level (data not shown). These moderate to strong correlations were mainly observed when the double hit group was included (Fig. 7B–H). However, including single insults in analyses resulted in no or only minor correlations (Suppl. Figure 8).

## Discussion

Inflammation during the perinatal period was suggested to sensitize the developing brain, making it more vulnerable to secondary harmful events in the early postnatal period [14, 16, 22, 31]. Here, we established a double-hit model of prenatal inflammation and postnatal hyperoxia, mimicking the clinical pathology observed in preterm infants diagnosed with WMI. By performing an in-depth characterisation, systematically including single insult groups, we identified distinct differences between single hits and the combination. Additive and synergistic effects were evident in brain volume, myelination deficits and neuroinflammatory responses -the major hallmarks of WMI- making this injury model particularly suitable to investigate novel therapeutic approaches for the treatment of WMI.

Besides inflammation, hyperoxia is a main perinatal risk factor for the development of WMI [14]. Our findings reveal that combining prenatal inflammation and postnatal hyperoxia resulted in reduced body weight and length, both typically associated with growth restriction, which increases the risk of WMI [14, 67, 68]. In support of this, correlation analyses of our experimental findings revealed a significant positive correlation between body weight and brain volume. Interestingly, these alterations were accompanied by increased serum leptin levels at term equivalent age, predominantly induced by prenatal LPS and further enhanced by postnatal hyperoxia. Leptin is a proteohormone that plays a crucial role in important physiological functions including regulation of immune responses, haematopoiesis, angiogenesis, bone formation, and wound healing [69]. Notably, higher leptin levels have been reported in umbilical cord blood of mothers with pre-eclampsia, which is often linked to growth restriction in the offspring [70], supporting the present findings of reduced body weight and length. However, increased leptin levels seem to contradict previous studies, showing lower levels in preterm babies [71]. These levels typically rise around term equivalent age, which is the time point of analyses in this study. It is important to note that elevated leptin levels are associated with higher fat accumulation at term-corrected age in preterm born babies [71]. The fat content, changes of Leptin expression in the brain and potential further metabolic alterations induced by the present injury model need to be investigated in future studies.

Very preterm and low birth weight infants exhibit smaller brain volumes at term equivalent age compared to term born babies, which are associated with impaired neurocognitive outcomes [72, 73]. Our study also revealed reductions in brain volume and deep cortical layer thickness, particularly at the hippocampal level, in pups exposed to prenatal inflammation and postnatal hyperoxia. Interestingly, slight effects on brain volume and TBR1 cortical layer thickness following prenatal LPS exposure were exacerbated by additional hyperoxia, indicating a synergistic impact. This was further supported by a significant positive correlation between brain volume and cortical thickness, when including the combined treatment group, while no correlation was observed when including single treatment groups. These results emphasise the concept that prenatal inflammation sensitise the immature brain to secondary postnatal insults, as suggested in context of additional postnatal hypoxia [30]. Furthermore, experimental findings as well as clinical evaluations in preterm infants, showed a decline in cortical thickness in early childhood and adolescence [74–76]. TBR1 is crucial for neocortical layer VI differentiation, and any alterations may negatively modulate brain development [77, 78]. Consistent with this Wu et al. have shown, that maternal inflammation induced by application of 75 µg/ kg LPS at embryonic day 14.5, resulted in a thinner TBR1^+^ cortical plate at embryonic day 18.5, which was associated with impaired neurodevelopment [79]. Using a higher dose of LPS (100 µg/ kg), we observed a slight thinning of the TBR1^+^ deep cortical layer after a single prenatal LPS application, which might be related to differences in LPS application time points, LPS serotypes and analysis time points. Nevertheless, the combination with the second hit of hyperoxia led to a significant reduction in the TBR1^+^ cell layer, which is supposed to be particularly important for proper neurodevelopment [80, 81]. Therefore, future studies should investigate long-term neurodevelopmental outcomes in the present injury model, which was beyond the scope of this study.

The most prominent pathological hallmarks of preterm birth related WMI are degeneration and altered differentiation of oligodendrocytes, resulting in impaired myelination [5, 12]. Consistent with our previous work [13, 21, 26], postnatal hyperoxia induced oligodendrocyte degeneration and reduced the number of mature CC1^+^ oligodendrocytes at P5. While prenatal LPS had no impact on the amount of degenerating and mature oligodendrocytes at P5, a reduction of mature oligodendrocytes was observed at term-equivalent age (P11). These results indicate different target mechanisms for each insult. A maturation deficit mediated by prenatal inflammation and additional degeneration induced by postnatal hyperoxia. Oligodendrocyte degeneration and maturation deficits have also been observed in other injury models, involving prenatal or postnatal inflammation combined with postnatal hypoxia or hyperoxia [13, 30, 82]. Brehmer et al. found that LPS application alone did not affect the oligodendrocyte degeneration compared to postnatal hyperoxia but did result in a maturation deficit. However, the combination of both insults had no additive effects on oligodendrocyte degeneration or maturation [13]. While Tilborg et al. detected no alterations in oligodendrocyte degeneration, they noted a reduced differentiation at P5 in the setting of prenatal inflammation combined with postnatal hypoxia [30]. Renz et al. 2022 reported both, an increased degeneration and impaired oligodendrocyte differentiation in a model of postnatal inflammation plus hypoxia [82]. The variability in findings may be attributed to differences in time points of the insults (i.e. prenatal vs. postnatal LPS and hypoxia at P4 vs P2), and different analysis time points (P3 vs. P5) [10, 30, 43, 82]. Comparing our results with findings from Tilborg et al., i.e. prenatal inflammation plus postnatal hypoxia, indicate differences between the impact of the second postnatal insult, with hyperoxia leading to more pronounced oligodendrocyte cell death than postnatal hypoxia [30]. Tilborg et al. demonstrated that prenatal LPS exposure results in a slight MBP reduction, while postnatal hypoxia has no significant impact on myelination. However, the combination of both insults leads to a marked hypomyelination at P18 [30]. Similarly, we observed the most pronounced effects by the combination of prenatal LPS and postnatal hyperoxia on myelination at P11. Decreased MBP expression level was accompanied by reduced expression of other myelin components, i.e. CNPase and MAG, the latter being particularly important for compact myelin formation and glia-axon interactions [83–85]. Ultrastructural analysis of myelin in MAG deficient mice (MAG^−/−^) revealed abnormal myelin structures and a reduction in axon calibre [86, 87], similar to findings from neonatal brain injury, caused by a single hit of postnatal hyperoxia [27, 59]. In support of this, the present results from structural analysis of MBP fibres, revealing reduced fibre lengths and intersections, suggest abnormalities in myelin formation after prenatal inflammation plus postnatal hyperoxia. Similar results were obtained in the combined model of prenatal inflammation and postnatal hypoxia [30].

Myelin formation is a complex process, depending on the support of different cell types, such as microglia. Microglia are essential for healthy compact myelin formation, preventing hypermyelination under physiological conditions [63]. However, under pathophysiological conditions, like WMI, microglia can also disturb myelination processes, by releasing pro-inflammatory cytokines [7, 88]. In line with previous findings in experimental models of maternal immune activation [89, 90], prenatal LPS led to microglia activation demonstrated by an increased proportion of CD68^+^ microglia from dams that were exposed to prenatal LPS. Interestingly, while postnatal hyperoxia did not modulate the percentage of CD68^+^ cells, it exacerbated the effect of prenatal LPS exposure. Morphological analysis of elongation revealed that prenatal LPS combined with postnatal hyperoxia led to a reduced cellular elongation. Microglia elongation is an indicator of protective and plasticity-promoting microglia, important for maintaining CNS homeostasis and secreting anti-inflammatory cytokines [91] Therefore, reduced elongation supports our findings obtained from CD68 staining and underscores that the double hit induces pronounced microglia activation. Our results underline the idea that perinatal inflammation sensitise the immature brain to a second insult, as previously shown in other multiple hit models, combining for example prenatal LPS exposure with postnatal hypoxia [10, 30, 31] or postnatal LPS exposure with hypoxia–ischemia [92–94]. In accordance with an increased microglia activation observed at P11, but not at P5, the combination of prenatal LPS and postnatal hyperoxia resulted in a pronounced upregulation of the pro-inflammatory cytokines IL6 and TNFα at P11. Yellowhair and colleagues have shown that the pro-inflammatory chemokine (C-X-C-motif) ligand 1 (CXCL1) and its receptor CXCR2 are critical for microglia recruitment and activation in an experimental model of chorioamnionitis, induced by the combination of in utero hypoxia and intra-amniotic inflammation, leading to neuroinflammation and microstructural alterations in the grey and white matter at P15 [95]. Our findings of pronounced microglia activation and elevated pro-inflammatory cerebral cytokine levels at P11, both associated with impaired myelination and brain growth, support the importance of microglia and delayed inflammatory processes in the development of perinatal brain injury [96]. Notably, in addition to upregulated pro-inflammatory cytokines, a significant increase in the anti-inflammatory cytokine IL10 was observed in the brain of pups exposed to the double hit. This may reflect the physiologically conserved mechanisms to counteract the strong pro-inflammatory responses, thereby protecting the organism from excessive inflammation, especially in the perinatal period [97–100].

Whether regulations of CNS immune responses were associated with alterations in the peripheral immune system was investigated by multiplex cytokine analysis in serum samples. In contrast to elevated cytokine expression in the brain, a pronounced reduction of cytokine levels was observed in the periphery. Similar discrepancies were also reported in a clinical study, demonstrating that preterm babies with MRI-defined WMI had higher levels of IL6, TNFα and IL10 in the cerebrospinal fluid, which were not reflected in plasma samples [101]. The underlying mechanisms remain speculative, but different cellular sources may play a critical role. While peripheral blood cytokines are most likely derived from circulating leukocytes, a variety of CNS resident cells produce both pro- and anti-inflammatory mediators in response to injury in the brain [102, 103]. Even though the single insult of postnatal hyperoxia for 24 h did not result in peripheral immune cell infiltration [21], far less is known about potential effects induced by prenatal LPS injection on blood brain barrier integrity and peripheral leukocyte entry. Future studies will need to thoroughly analyse the role of peripheral immune cells in the newly established experimental model to better understand the inflammatory pathways associated with preterm WMI. Even though our results suggest an interplay between central and peripheral immune activation in the pathogenesis of WMI, data need to be interpreted with caution, considering different methods of cytokine analyses (i.e. protein analysis in serum *vs*. mRNA expression analyses in brain samples). Protein expression analyses in brain tissue lysates will be needed. Furthermore, in an experimental model of chorioamnionitis combining in utero hypoxia with intra-amniotic inflammation, long-lasting alterations in peripheral immune responses have been described until the age of P120, suggesting a persistent state of immune dysregulation that may amplify vulnerability to secondary insults later in life [104]. These findings not only support our observation of altered systemic immune responses in the combined setting of prenatal inflammation and postnatal hyperoxia, but also highlight that perinatal insults have long-lasting effects far beyond the neonatal period. This was also nicely shown in models combining in utero hypoxia–ischemia and intra-amniotic inflammation resulting in long-term impaired brain development (i.e. impaired myelination and axon integrity) and motor dysfunction [105]. This is supported by clinical observations showing an association between neurodevelopmental outcome and brain volume and/or myelination deficits [106, 107]. We observed a positive correlation between myelination and brain volume at term-equivalent age. However, long-term neurodevelopmental outcome needs to be assessed in future work. In the present work, we focused on early pathophysiological processes and their interrelations, which was nicely presented by correlation analyses of our findings. For instance, microglial activation appears to impair brain development, as evidenced by significant negative correlations with brain volume and myelination. Together, these findings support the harmful role of neuroinflammation in WMI pathophysiology, as supported by previous studies [64, 108]. Nevertheless, in view of the afore mentioned reports, long-term assessment of peripheral and cerebral immune responses in association with microstructural alterations in the white matter and neurobehavioral deficits need to be explored in future studies.

Despite enormous efforts to mimic WMI experimentally, single insults can hardly reflect the multifactorial origin of preterm birth related WMI. In the present work, we combined the most common risk factors, i.e. prenatal inflammation and postnatal hyperoxia, resulting in major hallmarks of WMI in preterm infants, like reduced brain volume, thinner deep cortical layer and myelination deficits. As potential underlying mechanisms, we identified oligodendrocyte degeneration and impaired maturation associated with a pronounced neuroinflammatory response. A particular strength of the present work is the inclusion of single insults, allowing conclusions about neurobiological effects induced by each single insult and additive/synergistic effects by the combination of both insults. For example, preconditioning with prenatal LPS sensitizes the neonatal brain to the second hit of postnatal hyperoxia, resulting in more severe grey matter reduction associated with enhanced myelination deficits, increased microglia activation and inflammatory cytokine expression compared to each single insult. This experimental setup may serve as a novel translational model with high clinical relevance to investigate new treatment options such as stem cell-based therapies.

## Supplementary Information

Supplementary Material 1

## Data Availability

No datasets were generated or analysed during the current study.

## Abbreviations

CC1: Adenomatous polyposis coli, clone CC1
CD: Cluster of differentiation
CNPase: 2’, 3’- Cyclic nucleotide 3’-phosphodiesterase
DAPI: 4’, 6-Diamidin-2-phenylindol
EoP: Encephalopathy of prematurity
Iba1: Ionized calcium binding adaptor protein 1
IL: Interleukin
Ki67: Antigen Kiel 67
PFA: Paraformaldehyde
LPS: Lipopolysaccharide
MAG: Myelin associated glycoprotein
MBP: Myelin basic protein
NaCl: Sodium chloride
Olig2: Oligodendrocyte transcription factor 2
PCR: Polymerase chain reaction
ROI: Region of interest
TBR1: T-box transcription factor 1
TNFα: Tumor necrosis factor alpha
WMI: White matter injury
