# Supplementary material for: Prenatal inflammation exacerbates hyperoxia-induced neonatal brain injury
Source: J Neuroinflammation. 2025 Feb 28;22:57. doi: 10.1186/s12974-025-03389-4 (PMC11871844; doi:10.1186/s12974-025-03389-4)
Supplement: Supplementary file 1 — Supplementary Material 1 [file 12974_2025_3389_MOESM1_ESM.pdf]

Additional File1: Supplementary Figures and Tables

Prenatal inflammation exacerbates hyperoxia-induced neonatal brain injury

Meray Serdar, Kay-Anja Walther, Markus Gallert, Karina Kempe, Stefanie Obst, Nicole Labusek, Ralf Herrmann, Josephine Herz, Ursula Felderhoff-Müser and Ivo Bendix[#]

Department of Paediatrics I, Neonatology and Experimental perinatal Neurosciences; Centre for Translational Neuro- and Behavioural Sciences (C-TNBS), University Hospital Essen, University Duisburg-Essen, Essen, Germany

#Correspondence to:

Ursula Felderhoff-Müser: ursula.felderhoff@uk-essen.de

Ivo Bendix: ivo.bendix@uk-essen.de

Supplementary Figures: 9

Supplementary Tables 4

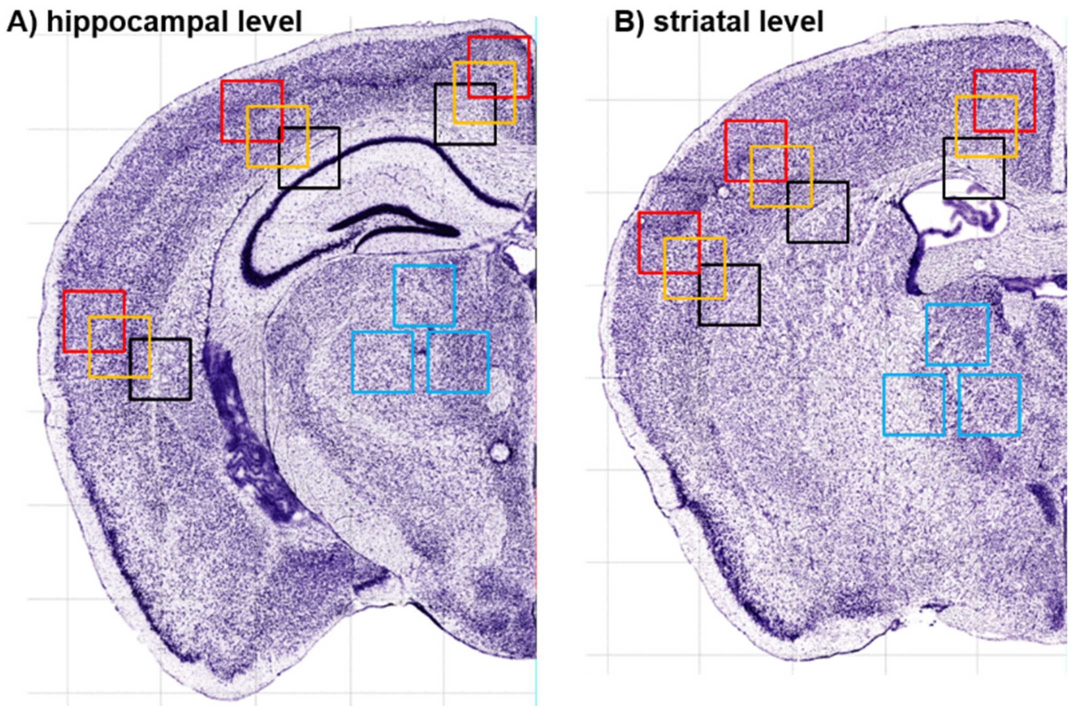

Supplement 1. Regions of interests analysed via immunohistochemistry. Oligodendrocyte degeneration, the quantity of mature oligodendrocytes, microglia activation (black boxes white matter, red boxes cortex, blue boxes thalamus in A and striatum in B) and myelination (yellow boxes) were assessed in the depicted areas at the hippocampal (A, 3.72±0.7 mm) and striatal (B, -0.6±0.3 mm) level.

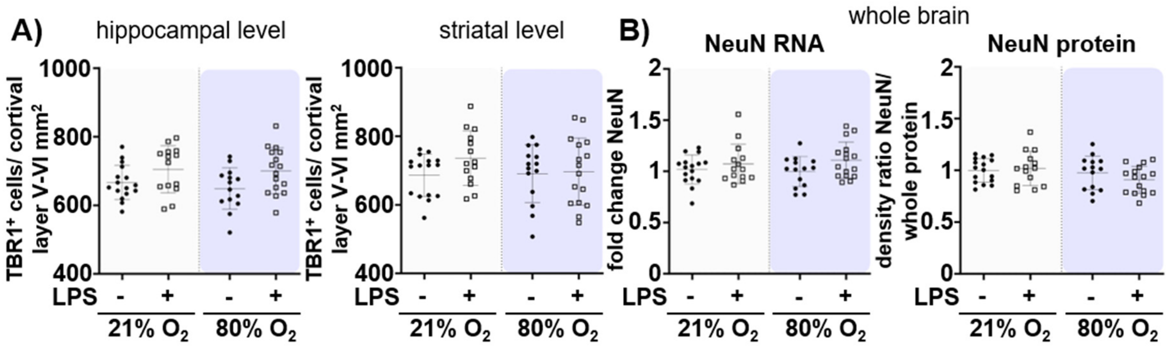

Supplement 2. Prenatal inflammation combined with postnatal hyperoxia does not affect the density of total neurons in the entire hemisphere and of TBR1⁺ neurons in the deep cortical layers. Following prenatal inflammation induced at E20, newborn pups were exposed to postnatal hyperoxia from P3 to P5 and analysed by real time PCR, western blot and immunohistochemistry at P11. A) The amount of $TBR1^{+}$ neurons was quantified via immunohistochemistry. Cell numbers were quantified and related to the total region of TBR1-positively stained areas in the deep cortical layers V and VI at the hippocampal (3.72±0.7 mm) and striatal level (-0.6±0.3 mm). B) As measure of total neuronal density, mRNA and protein expression of the pan-neuronal marker NeuN were quantified in tissue lysates of the entire hemisphere. n= 14-16 rats/ group.

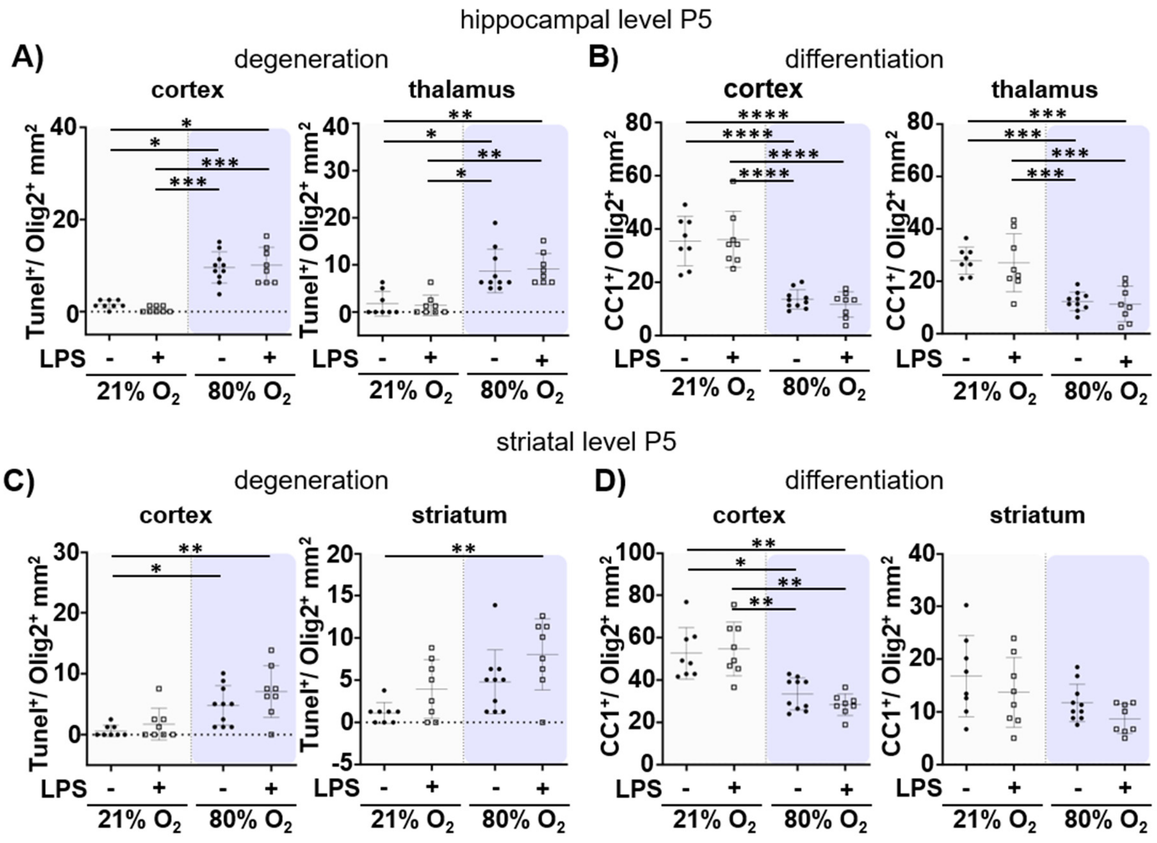

Supplement 3. Impact of prenatal inflammation and postnatal hyperoxia on oligodendrocyte degeneration and mature oligodendrocytes in the cortex, thalamus and striatum at P5. Pups from dams exposed to LPS followed by postnatal hyperoxia (80% O_2) at P3 for 48 h were analysed immediately after hyperoxia at P5. The number of degenerating and mature oligodendrocytes was analysed in the hippocampal (3.72±0.7 mm from bregma, A, B) and striatal (-0.6±0.3 mm from bregma, C, D) level. A), C) Oligodendrocyte degeneration was assessed by quantification of Tunel/Olig2 double positive cells. B), D) The number of mature oligodendrocytes was determined by quantification of by CC1/Olig2 double positive cells. n=8-10 rats/ group $^*p<0.05$, $^{**}p<0.01$, $^{***}p<0.001$, $^{****}p<0.0001$.

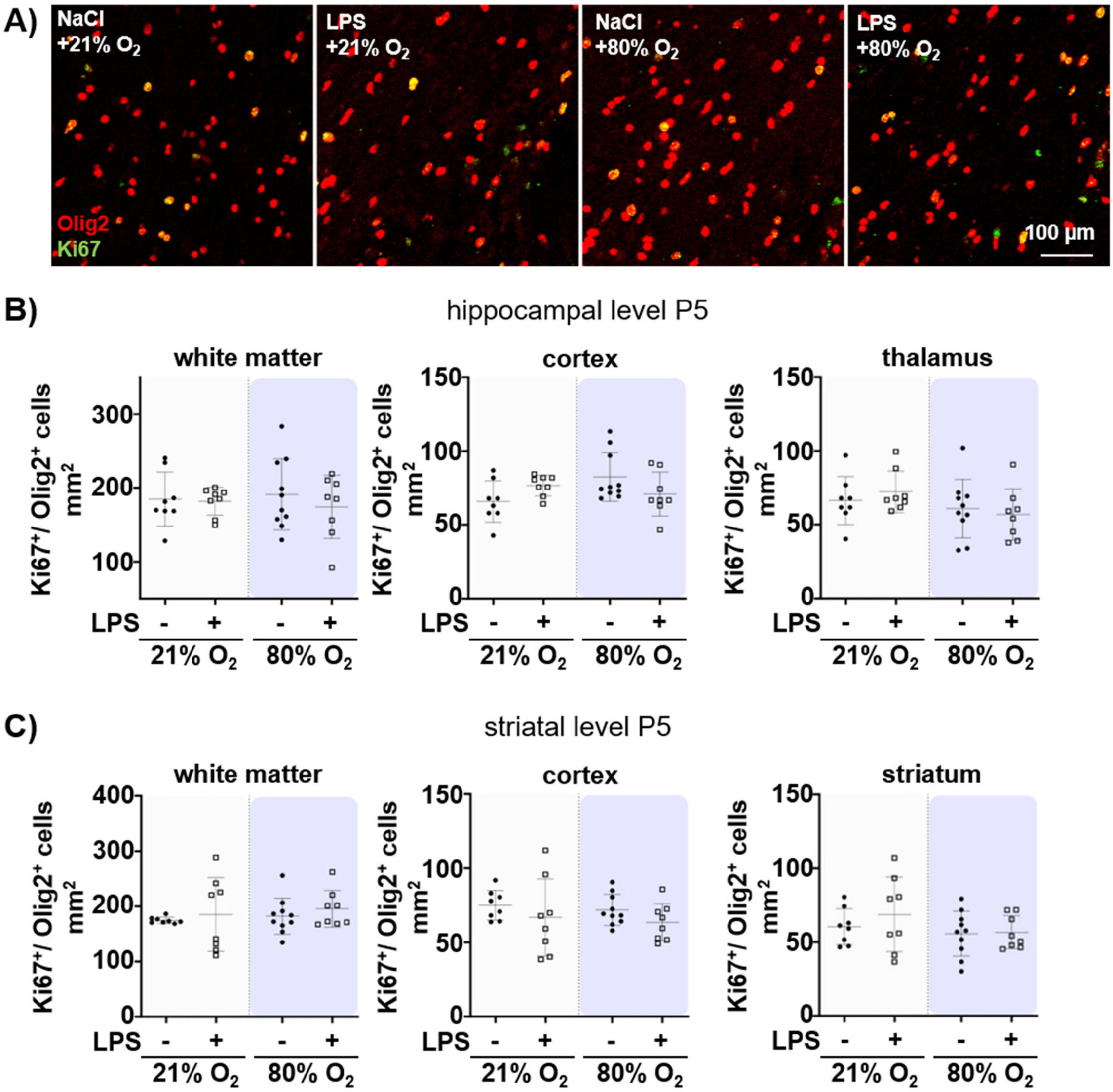

Supplement 4. Prenatal inflammation combined with postnatal hyperoxia does not alter oligodendrocyte proliferation at P5. Offspring of dams that received LPS injections at E20 were exposed to postnatal normoxia (21% O_2) or hyperoxia (80% O_2) from P3 to P5. Immediately after the second event, oligodendrocyte proliferation was investigated by Olig2 (red) Ki67 (green) staining. A) Representative image of Olig2/ Ki67 co-staining (yellow) in the capsula externa of the white matter region (Suppl. Fig. 1). The amount of double positive cells was quantified in the depicted brain regions at the hippocampal (3.72±0.7 mm, B) and striatal (-0.6 ± 0.3 mm, C) level. n= 8-10 rats/group.

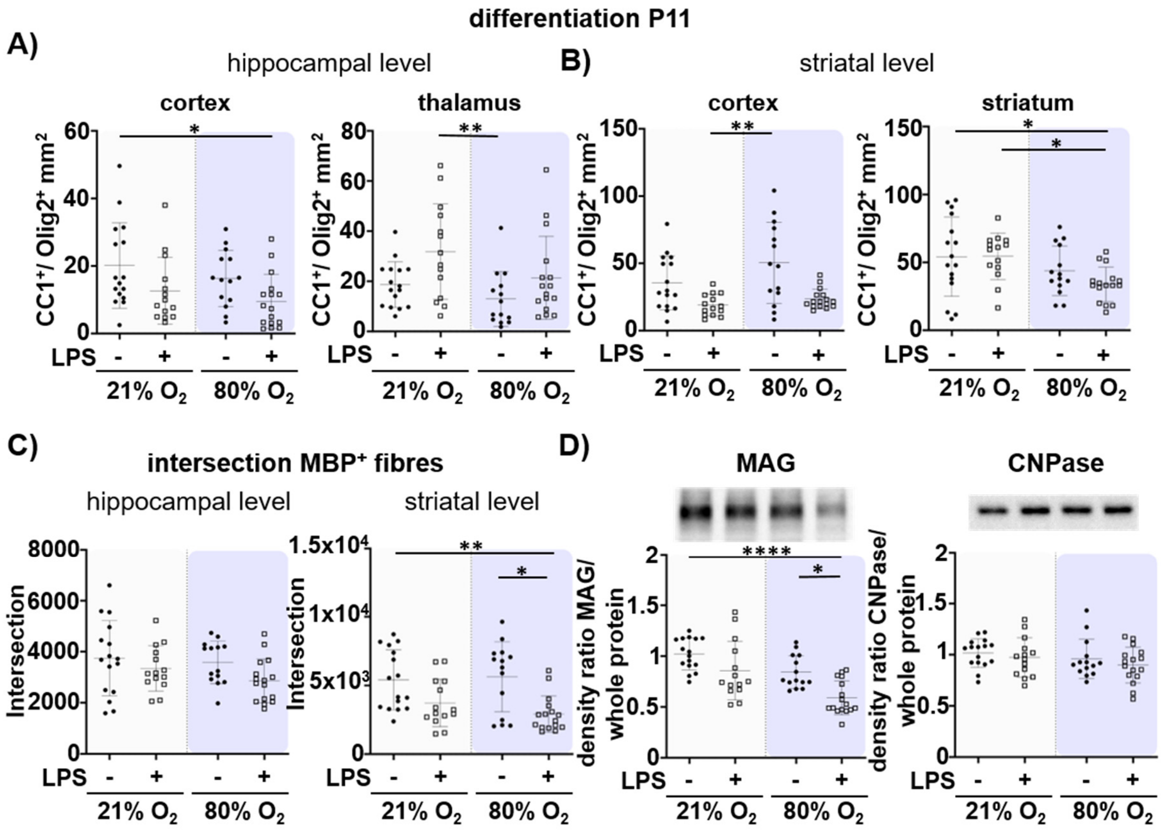

Supplement 5 Myelination is disturbed by postnatal hyperoxia alone or in combination with prenatal inflammation at P11. Oligodendrocyte differentiation and myelination were analysed 6 days post normoxia (21% O_2) or hyperoxia (80% O_2) via immunohistochemistry and western-blot. A, B) Differentiation of oligodendrocytes (CC1/ Olig2) was analysed at the hippocampal (3.72±0-7 mm, A) and striatal (-0.6±0.3 mm, B) level in cortex, thalamus and striatum. C) The amount of MBP positive fibre intersections was quantified by the adapted Diameter J plugin in the defined ROIs (Suppl. Fig. 1) at the hippocampal and striatal level. D) Protein expression of myelin proteins MAG and CNPase was quantified in whole hemisphere brain tissue lysates normalized to whole protein and the control (NaCl + 21% O_2). E) Correlation analysis of different outcome markers following maternal LPS and postnatal hyperoxia (LPS + 80% O_2). mRNA expression of MAG and MBP^+ fibre length (left), mRNA MAG and brain volume (middle), MBP^+ fibre length and brain volume (right), MBP^+ intersection and MBP^+ fibre length for hippocampal and striatal level (right). n=14-16 rats/ group. *$p<0.05$, **$p<0.01$, ****$p<0.0001$ (A-D).

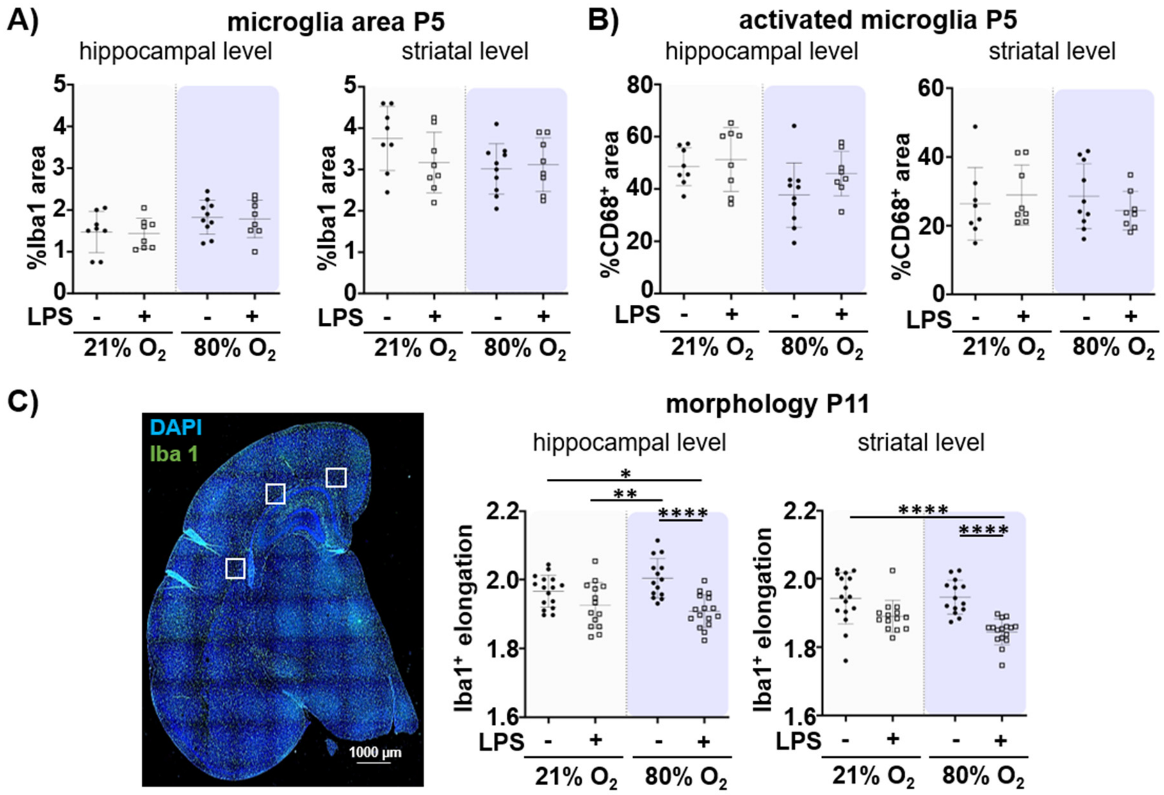

Supplement 6 Neither single hits of prenatal inflammation and postnatal hyperoxia nor the combination of both modulate microglia activation at P5. Pups from dams that received LPS injections at E20, which were additionally exposed to postnatal normoxia (21% O_2) or hyperoxia (80% O_2) between P3 and P5 were analysed immediately after hyperoxia via immunohistochemistry for Iba-1 and CD68. A) The percentage of Iba1 positive area was analysed in regions of the white matter (Suppl. Fig. 1) at the hippocampal (3.72±0.7 mm) and striatal (-0.6±0.3 mm) level. B) As a measure of microglia activation the percentage of $CD68^+$ area of the Iba-1 positively stained area was quantified. n= 8-10 rats/ group. C) Example confocal image of DAPI IBA1 staining. The white boxes mark the white matter used for quantification (left). Morphological changes, such as circularity, in $Iba1^+$ microglia were analysed by using the NIS-Software. n= 14-16 rats/ group. **p<0.01.

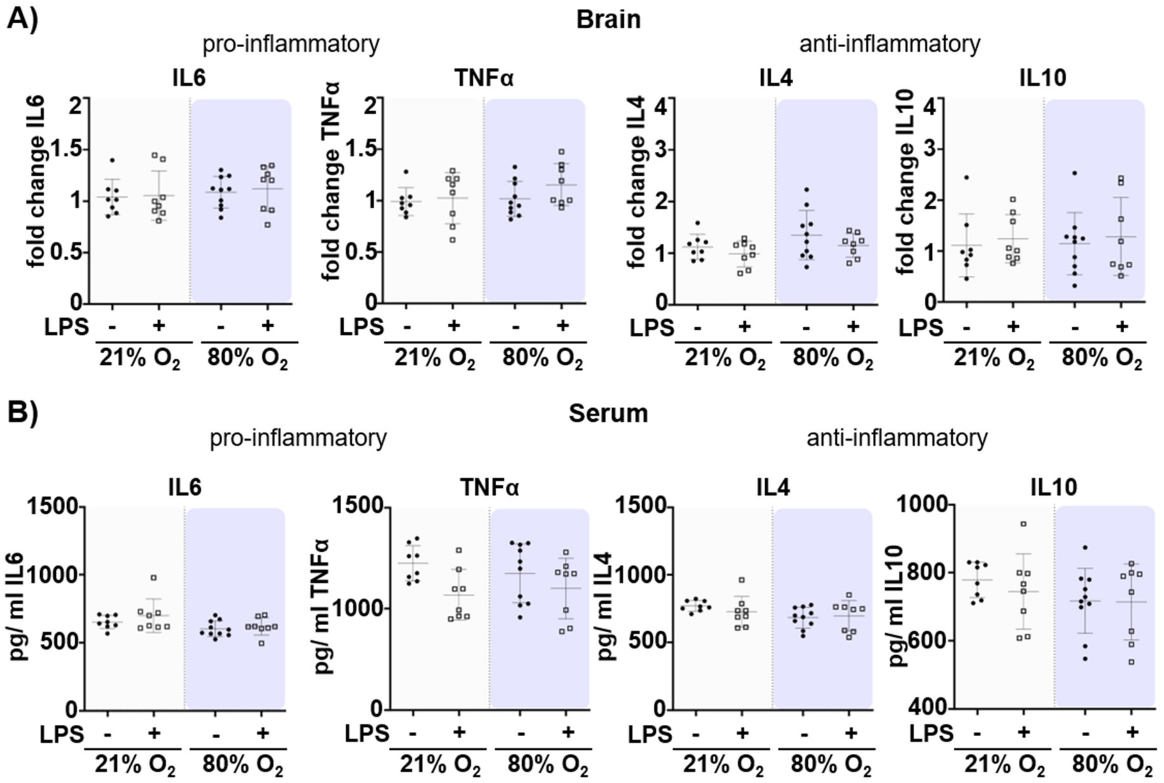

Supplement 7 Prenatal inflammation, postnatal hyperoxia and the combination of both do not regulate pro- and anti-inflammatory cytokine levels in brain and serum at P5. Pups from dams that received LPS injections at E20 were exposed to postnatal normoxia (21% O_2) or hyperoxia (80% O_2) between P3 and P5 were analysed immediately after hyperoxia at P5. A) The expression of pro- (IL6, TNF-α) and anti- (IL4, IL10) inflammatory cytokines was quantified in brain tissue lysates via real time PCR. B) Serum samples were analysed via multiplex protein expression analyses. n=8-10 rats/ group.

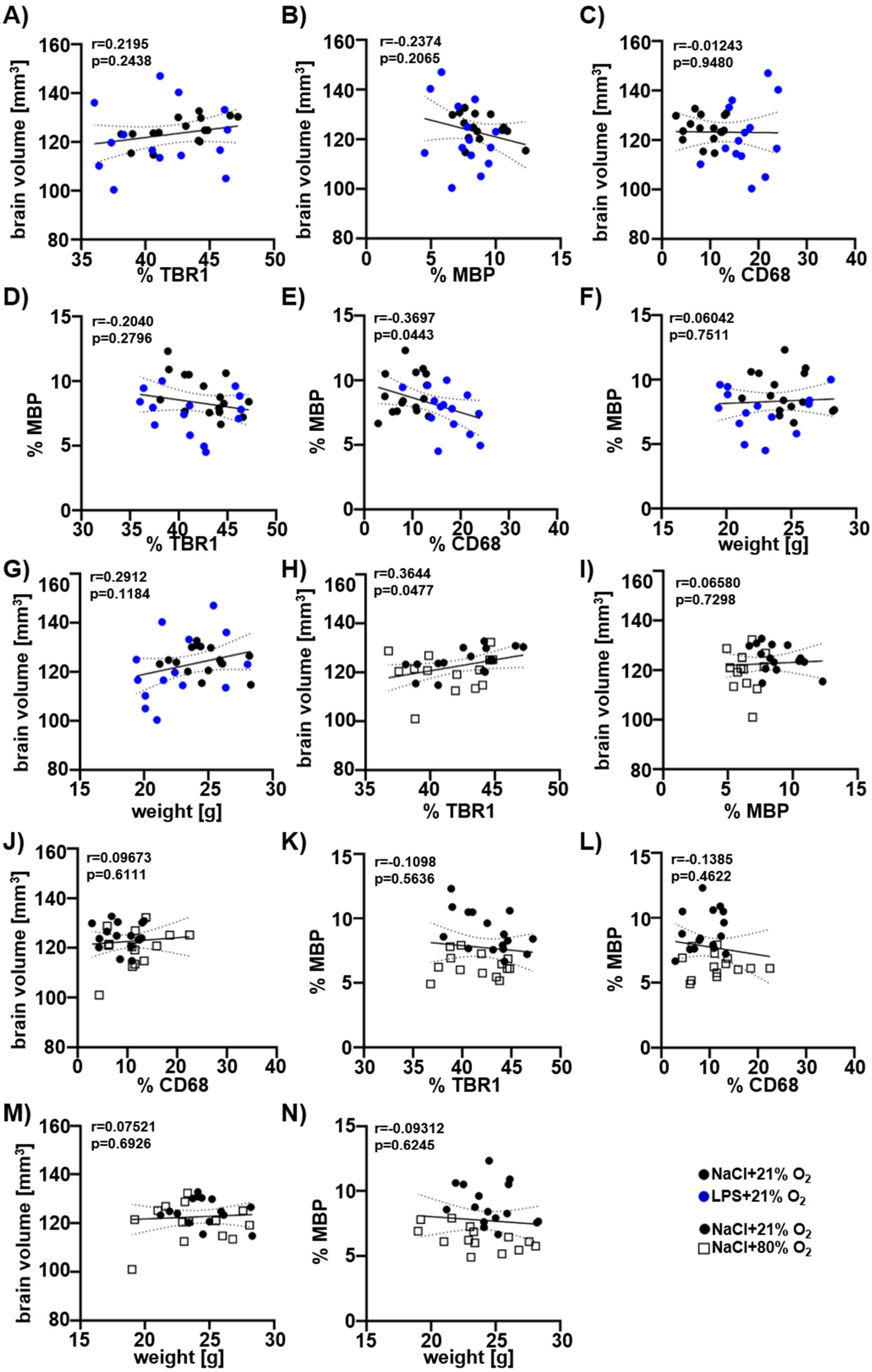
A)
r=0.2195
p=0.2438
brain volume [mm³]
% TBR1
B)
r=-0.2374
p=0.2065
brain volume [mm³]
% MBP
C)
r=-0.01243
p=0.9480
brain volume [mm³]
% CD68
D)
r=-0.2040
p=0.2796
% MBP
% TBR1
E)
r=-0.3697
p=0.0443
% MBP
% CD68
F)
r=0.06042
p=0.7511
% MBP
weight [g]
G)
r=0.2912
p=0.1184
brain volume [mm³]
weight [g]
H)
r=0.3644
p=0.0477
brain volume [mm³]
% TBR1
I)
r=0.06580
p=0.7298
brain volume [mm³]
% MBP
J)
r=0.09673
p=0.6111
brain volume [mm³]
% CD68
K)
r=-0.1098
p=0.5636
% MBP
% TBR1
L)
r=-0.1385
p=0.4622
% MBP
% CD68
M)
r=0.07521
p=0.6926
brain volume [mm³]
weight [g]
N)
r=-0.09312
p=0.6245
% MBP
weight [g]
NaCl+21% O2
LPS+21% O2
NaCl+21% O2
NaCl+80% O2

Supplement 8 Prenatal inflammation, postnatal hyperoxia and the combination of both do not regulate pro- and anti-inflammatory cytokine levels in brain and serum at P5. Pups from dams that received LPS injections at E20 followed by postnatal normoxia (21% O_2) or hyperoxia (80% O_2) between P3 and P5 were analysed at P11. A-N) Correlation analyses were performed for key features of WMI injury, i.e. body weight, brain volume, cortical thickness (% TBR1), myelination (% MBP) and microglial activation (% CD68) for the individual hits (LPS + 21% O_2 (A-G) and NaCl + 80% O_2 (H-N)) compared to the control. Pearson´s (parametric) or Spearmann´s rank correlation coefficient (non-parametric) (r) and the p-value were calculated for each correlation. n=14 rats/ group.

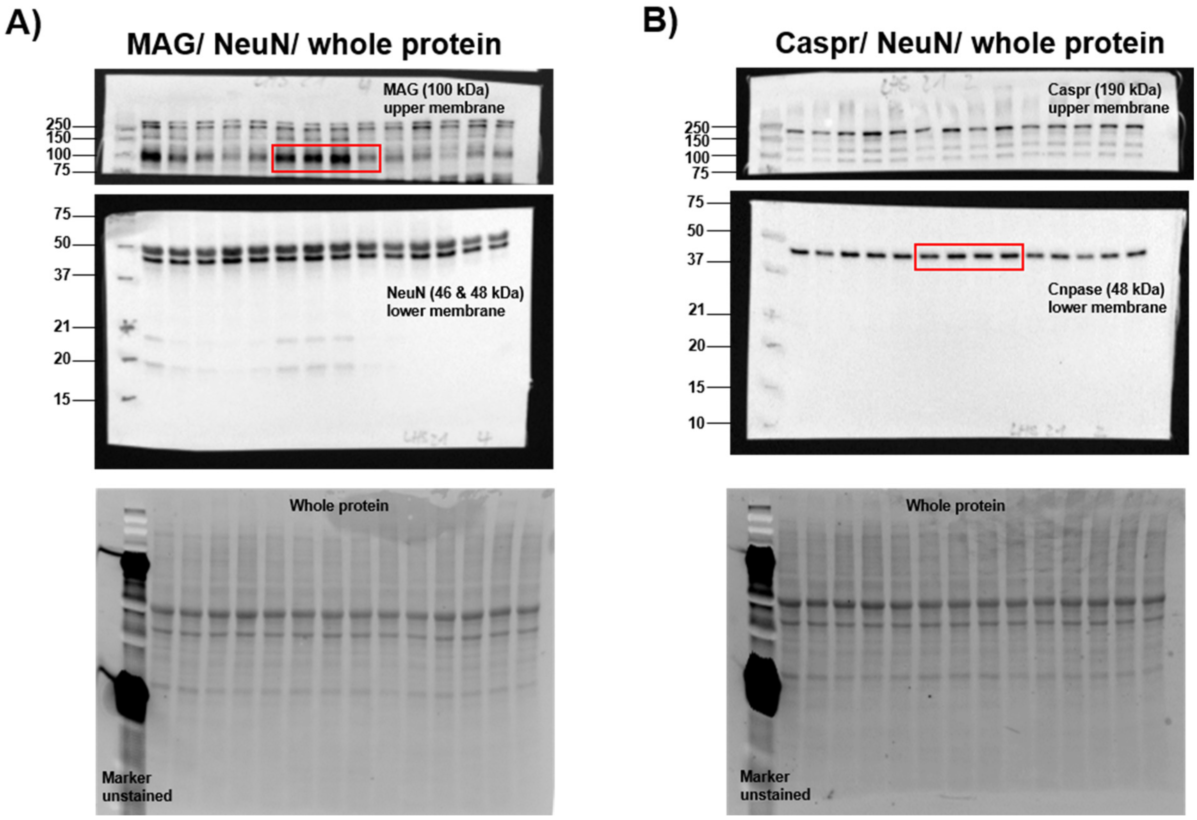

Supplement 9 Original full-length western blot. All images represent merged images of original western blot images and transmitted light images to visualize ladder bands (unstained marker). To avoid cross reactions of primary or secondary antibodies membranes were cut at approximately 75 kDa prior to antibody incubation and detection. A) Analysis of MAG and NeuN was performed on the same membrane B) Contactin associated Protein 1 (Caspr) and CNPase were detected on the same membrane. Protein expression was normalized to whole protein content (lower images in A and B). Caspr expression was not modulated by either insult and is therefore not shown in the manuscript. Red boxes indicate cropped regions used for illustrations in the main figures of the manuscript.

Table S1: TaqMan Assays for mRNA expression analysis

Gene	Assay ID
Interleukin 6 (IL-6)	Rn01410330_m1
Tumor necrosis factor alpha (TNF-α)	*Rn01525859_g1*
Interleukin 4 (IL-4)	Rn01456866
Interleukin 10 (IL-10)	Rn01483988_g1
Beta-2-microtubulin (B2M)	Rn00560865_m1

Table S2: Two-Way ANOVA results of data from main figures 1-3

**Figure 1**								
	**weight P5**		**weight P11**		**body length P11**		**leptin serum P11**	
**Interaction LPS x 80% O_2**	F (1, 90) = 0.1349	p= 0.7142 (n.s.)	F (1, 56) = 0.0705	p= 0.7915 (n.s.)	F (1, 56) = 1.510	p= 0.2243 (n.s.)	F (1, 30) = 6.383	p= 0.0170 (*)
**80% O_2**	F (1, 90) = 8.754	p= 0.0039 (**)	F (1, 56) = 3.920	p= 0.0526 (n.s.)	F (1, 56) = 1.097	p= 0.2993 (n.s.)	F (1, 30) = 1.506	p= 0.2293 (n.s.)
**LPS**	F (1, 90) = 6.927	p= 0.0100 (**)	F (1, 56) = 11.78	p= 0.0011 (**)	F (1, 56) = 23.87	p <0.0001 (****)	F (1, 30) = 30.42	p <0.0001 (****)
**Figure 2**								
	**brain volume**		**% TBR1 hippocampal level**		**% TBR1 striatal level**			
**Interaction LPS x 80% O_2**	F (1, 56) = 8.370	p= 0.0054 (**)	F (1, 56) = 4.843	p= 0.0319 (*)	F (1, 56) = 0.001	p= 0.9701 (n.s.)		
**80% O_2**	F (1, 56) = 22.79	p <0.0001 (****)	F (1, 56) = 8.435	p= 0.0053 (**)	F (1, 56) = 0.006	p= 0.9400 (n.s.)		
**LPS**	F (1, 56) = 17.68	p <0.0001 (****)	F (1, 56) = 9.568	p= 0.0031 (**)	F (1, 56) = 0.070	p= 0.7930 (n.s.)		
**Figure 3**								
	**degeneration P5 hippocampal level**		**degeneration P5 striatal level**		**differentiation P5 hippocampal level**		**differentiation P5 striatal level**	
**Interaction LPS x 80% O_2**	F (1, 30) = 2.519	p= 0.1229 (n.s.)	F (1, 30) = 2.177	p= 0.1505 (n.s.)	F (1, 30) = 0.041	p= 0.8418 (n.s.)	F (1, 29) = 2.300	p= 0.1402 (n.s.)
**80% O_2**	F (1, 30) = 65.53	p <0.0001 (****)	F (1, 30) = 30.11	p <0.0001 (****)	F (1, 30) = 23.38	p <0.0001 (****)	F (1, 29) = 12.51	p= 0.0014 (**)
**LPS**	F (1, 30) = 3.332	p= 0.0779 (n.s.)	F (1, 30) = 0.0027	p= 0.9591 (n.s.)	F (1, 30) = 0.034	p= 0.8546 (n.s.)	F (1, 29) = 0.781	p= 0.3841 (n.s.)
	**differentiation P11 hippocampal level**		**differentiation P11 striatal level**					
**Interaction LPS x 80% O_2**	F (1, 56) = 0.688	p= 0.4105 (n.s.)	F (1, 56) = 0.430	p= 0.5145 (n.s.)				
**80% O_2**	F (1, 56) = 9.457	p= 0.0032 (**)	F (1, 56) = 0.055	p= 0.8152 (n.s.)				
**LPS**	F (1, 56) = 3.838	p= 0.0551 (n.s.)	F (1, 56) = 15.76	p= 0.0002 (***)				

blue: significant interaction effect indicating a synergistic effect of prenatal LPS and postnatal hyperoxia

Table S3: Two-Way ANOVA results of data from main figures 4-6

**Figure 4**								
	**%MBP hippocampal level**		**%MBP striatal level**		**sum density hippocampal level**		**sum density striatal level**	
**Interaction LPS x 80% O_2**	F (1, 56) = 3.039	p= 0.0868 (n.s.)	F (1, 56) = 9.894	p= 0.0027 (**)	F (1, 52) = 0.661	p= 0.4200 (n.s.)	F (1, 56) = 2.558	p= 0.1154 (n.s.)
**80% O_2**	F (1, 56) = 97.21	p <0.0001 (****)	F (1, 56) = 107.2	p <0.0001 (****)	F (1, 52) = 42.10	p <0.0001 (****)	F (1, 56) = 101.0	p <0.0001 (****)
**LPS**	F (1, 56) = 17.16	p= 0.0001 (***)	F (1, 56) = 16.56	p= 0.0001 (***)	F (1, 52) = 16.73	p= 0.0002 (***)	F (1, 56) = 24.80	p <0.0001 (****)
	**mRNA MAG**		**mRNA CNPase**		**fibre length hippocampal level**		**fibre length striatal level**	
**Interaction LPS x 80% O_2**	F (1, 56) = 4.761	p= 0.0333 (*)	F (1, 56) = 10.57	p= 0.0019 (**)	F (1, 56) = 3.981	p= 0.0509 (n.s.)	F (1, 56) = 0.169	p= 0.1685 (n.s.)
**80% O_2**	F (1, 56) = 13.09	p= 0.0006 (***)	F (1, 56) = 11.95	p= 0.0010 (**)	F (1, 56) = 0.5854	p= 0.4474 (n.s.)	F (1, 56) = 0.052	p= 0.8199 (n.s.)
**LPS**	F (1, 56) = 2.572	p= 0.1144 (n.s.)	F (1, 56) = 3.572	p= 0.0639 (n.s.)	F (1, 56) = 29.41	p <0.0001 (****)	F (1, 56) = 18.74	p <0.0001 (****)
**Figure 5**								
	**%Iba1 hippocampal level**		**%Iba1 striatal level**		**%CD68 hippocampal level**		**%CD68 striatal level**	
**Interaction LPS x 80% O_2**	F (1, 56) = 10.46	p= 0.0020 (**)	F (1, 56) = 1.401	p= 0.2416 (n.s.)	F (1, 56) = 2.277	p= 0.1369 (n.s.)	F (1, 56) = 14.47	p= 0.0004 (***)
**80% O_2**	F (1, 56) = 5.262	p= 0.0256 (*)	F (1, 56) = 0.267	p= 0.6072 (n.s.)	F (1, 56) = 10.66	p= 0.00019 (**)	F (1, 56) = 1.931	p= 0.1701 (n.s.)
**LPS**	F (1, 56) = 2.395	p= 0.1274 (n.s.)	F (1, 56) = 24.20	p <0.0001 (****)	F (1, 56) = 49.94	p <0.0001 (****)	F (1, 56) = 39.47	p <0.0001 (****)
**Figure 6**								
	**mRNA IL 6**		**mRNA TNFα**		**mRNA IL4**		**mRNA IL10**	
**Interaction LPS x 80% O_2**	F (1, 56) = 8.410	p= 0.0053 (**)	F (1, 56) = 5.699	p= 0.0204 (*)	F (1, 56) = 2.268	p= 0.1377 (n.s.)	F (1, 56) = 0.571	p= 0.5714 (n.s.)
**80% O_2**	F (1, 56) = 13.68	p= 0.0005 (***)	F (1, 56) = 25.87	p <0.0001 (****)	F (1, 56) = 1.747	p= 0.1916 (n.s.)	F (1, 56) = 0.355	p= 0.5537 (n.s.)
**LPS**	F (1, 56) = 40.48	p <0.0001 (****)	F (1, 56) = 0.0873	p= 0.7687 (n.s.)	F (1, 56) = 7.746	p= 0.0073 (**)	F (1, 56) = 27.41	p <0.0001 (****)
	**serum IL 6**		**serum TNFα**		**serum IL4**		**serum IL10**	
**Interaction LPS x 80% O_2**	F (1, 28) = 1.805	p= 0.1899 (n.s.)	F (1, 30) = 1.150	p= 0.2921 (n.s.)	F (1, 30) = 1.593	p= 0.2166 (n.s.)	F (1, 30) = 0.307	p= 0.5835 (n.s.)
**80% O_2**	F (1, 28) = 11.24	p= 0.0023 (**)	F (1, 30) = 4.199	p= 0.0493 (*)	F (1, 30) = 14.70	p= 0.0006 (***)	F (1, 30) = 15.05	p= 0.0005 (***)
**LPS**	F (1, 28) = 2.205	p= 0.1487 (n.s.)	F (1, 30) = 3.725	p= 0.0631 (n.s.)	F (1, 30) = 11.04	p= 0.0024 (**)	F (1, 30) = 1.393	p= 0.2472 (n.s.)

blue: significant interaction effect indicating a synergistic effect of prenatal LPS and postnatal hyperoxia

Table S4: Two-Way ANOVA results of data from Supplement 3, 5 and 6

**Supplement 3**								
	**degeneration cortex P5 hippocampal level**		**degeneration thalamus P5 hippocampal level**		**differentiation cortex P5 hippocampal level**		**differentiation thalamus P5 hippocampal level**	
**Interaction LPS x 80% O_2**	F (1, 30) = 0.756	p= 0.3914 (n.s.)	F (1, 30) = 0.105	p= 0.7485 (n.s.)	F (1, 30) = 0.245	p= 0.2454 (n.s.)	F (1, 30) = 0.002	p= 0.9684 (n.s.)
**80% O_2**	F (1, 30) = 90.85	p <0.0001 (****)	F (1, 30) = 39.47	p <0.0001 (****)	F (1, 30) = 81.53	p <0.0001 (****)	F (1, 30) = 41.72	p <0.0001 (****)
**LPS**	F (1, 30) = 0.105	p= 0.1050 (n.s.)	F (1, 30) = 0.003	p= 0.9573 (n.s.)	F (1, 30) = 0.068	p= 0.7961 (n.s.)	F (1, 30) = 0.144	p= 0.7066 (n.s.)
	**degeneration cortex P5 striatal level**		**degeneration striatum P5 striatal level**		**differentiation cortex P5 striatal level**		**differentiation striatum P5 striatal level**	
**Interaction LPS x 80% O_2**	F (1, 30) = 0.329	p= 0.5706 (n.s.)	F (1, 30) = 0.0304	p= 0.8627 (n.s.)	F (1, 30) = 1.108	p= 0.3010 (n.s.)	F (1, 30) = 0.001	p= 0.9801 (n.s.)
**80% O_2**	F (1, 30) = 20.77	p <0.0001 (****)	F (1, 30) = 10.99	p= 0.0024 (**)	F (1, 30) = 45.69	p <0.0001 (****)	F (1, 30) = 7.205	p= 0.0177 (*)
**LPS**	F (1, 30) = 2.656	p= 0.1136 (n.s.)	F (1, 30) = 6.707	p= 0.0147 (*)	F (1, 30) = 0.198	p= 0.6599 (n.s.)	F (1, 30) = 2.645	p= 0.1144 (n.s.)
**Supplement 5**								
	**differentiation cortex P11 hippocampal level**		**differentiation thalamus P11 hippocampal level**		**differentiation cortex P11 striatal level**		**differentiation striatum P11 striatal level**	
**Interaction LPS x 80% O_2**	F (1, 56) = 0.018	p= 0.8942 (n.s.)	F (1, 56) = 0.414	p= 0.5226 (n.s.)	F (1, 56) = 1.192	p= 0.2795 (n.s.)	F (1, 56) = 0.957	p= 0.3321 (n.s.)
**80% O_2**	F (1, 56) = 1.839	p= 0.1805 (n.s.)	F (1, 56) = 4.676	p= 0.0349 (*)	F (1, 56) = 4.051	p= 0.0490 (*)	F (1, 56) = 8.631	p= 0.0048 (**)
**LPS**	F (1, 56) = 7.678	p= 0.0076 (**)	F (1, 56) = 8.372	p= 0.0054 (**)	F (1, 56) = 20.22	p <0.0001 (****)	F (1, 56) = 0.851	p= 0.3603 (n.s.)
	**Intersection hippocampal level**		**Intersection striatal level**		**WB MAG**		**WB CNPase**	
**Interaction LPS x 80% O_2**	F (1, 56) = 0.351	p= 0.5560 (n.s.)	F (1, 56) = 1.024	p= 0.3158 (n.s.)	F (1, 56) = 0.806	p= 0.3732 (n.s.)	F (1, 56) = 0.030	p= 0.8634 (n.s.)
**80% O_2**	F (1, 56) = 1.357	p= 0.2490 (n.s.)	F (1, 56) = 0.320	p= 0.5740 (n.s.)	F (1, 56) = 18.99	p <0.0001 (****)	F (1, 56) = 2.102	p= 0.1527 (n.s.)
**LPS**	F (1, 56) = 4.184	p= 0.0455 (*)	F (1, 56) = 18.23	p <0.0001 (****)	F (1, 56) = 16.23	p <0.0001 (****)	F (1, 56) = 1.308	p= 0.2576 (n.s.)
**Supplement 6**								
	**%Iba1 P5 hippocampal level**		**%CD68 P5 hippocampal level**		**%Iba1 P5 striatal level**		**%CD68 P5 striatal level**	
**Interaction LPS x 80% O_2**	F (1, 30) = 0.001	p= 0.9700 (n.s.)	F (1, 30) = 0.572	p= 0.4552 (n.s.)	F (1, 30) = 2.039	p= 0.1637 (n.s.)	F (1, 30) = 1.223	p= 0.2776 (n.s.)
**80% O_2**	F (1, 30) = 5.587	p= 0.0248 (*)	F (1, 30) = 5.098	p= 0.0314 (*)	F (1, 30) = 2.770	p= 0.1064 (n.s.)	F (1, 30) = 0.149	p= 0.7026 (n.s.)
**LPS**	F (1, 30) = 0.062	p= 0.8054 (n.s.)	F (1, 30) = 2.299	p= 0.1400 (n.s.)	F (1, 30) = 1.035	p= 0.3170 (n.s.)	F (1, 30) = 0.076	p= 0.7862 (n.s.)
	**elongation P11 hippocampal level**		**elongation P11 striatal level**					
**Interaction LPS x 80% O_2**	F (1, 56) = 3.808	p= 0.0560 (n.s.)	F (1, 56) = 3.013	p= 0.0881 (n.s.)				
**80% O_2**	F (1, 56) = 0.5624	p= 0.4565 (n.s.)	F (1, 56) = 2.228	p= 0.1412 (n.s.)				
**LPS**	F (1, 56) = 23.54	p <0.0001 (****)	F (1, 56) = 29.74	p <0.0001 (****)				
